# Sulforaphane suppresses the activity of sterol regulatory element-binding proteins (SREBPs) by promoting SREBP precursor degradation

**DOI:** 10.1038/s41598-022-12347-6

**Published:** 2022-05-24

**Authors:** Shingo Miyata, Manami Kodaka, Akito Kikuchi, Yuki Matsunaga, Kenta Shoji, Yen-Chou Kuan, Masamori Iwase, Keita Takeda, Ryo Katsuta, Ken Ishigami, Yu Matsumoto, Tsukasa Suzuki, Yuji Yamamoto, Ryuichiro Sato, Jun Inoue

**Affiliations:** 1 Department of Applied Biological Chemistry, Graduate School of Agricultural and Life Sciences, The University of Tokyo, Tokyo, 113-8657 Japan; 2 Department of Agricultural Chemistry, Faculty of Applied Biosciences, Tokyo University of Agriculture, Tokyo, 156-8502 Japan; 3 Department of Chemistry for Life Sciences and Agriculture, Tokyo University of Agriculture, Tokyo, 156-8502 Japan; 4 Present Address: Department of Horticulture and Landscape Architecture, College of Bioresources and Agriculture, National Taiwan University, Taipei, Taiwan

**Keywords:** Proteolysis, Transcription

## Abstract

Sterol regulatory element-binding proteins (SREBPs) are transcription factors that regulate various genes involved in cholesterol and fatty acid synthesis. In this study, we describe that naturally occurring isothiocyanate sulforaphane (SFaN) impairs fatty acid synthase promoter activity and reduces SREBP target gene (e.g., fatty acid synthase and acetyl-CoA carboxylase 1) expression in human hepatoma Huh-7 cells. SFaN reduced SREBP proteins by promoting the degradation of the SREBP precursor. Amino acids 595–784 of SREBP-1a were essential for SFaN-mediated SREBP-1a degradation. We also found that such SREBP-1 degradation occurs independently of the SREBP cleavage-activating protein and the Keap1-Nrf2 pathway. This study identifies SFaN as an SREBP inhibitor and provides evidence that SFaN could have major potential as a pharmaceutical preparation against hepatic steatosis and obesity.

## Introduction

Recently, a steady increase has been observed in the number of people suffering from lifestyle-related diseases, such as obesity, dyslipidemia, and type II diabetes^1^. The development of metabolic diseases is caused mainly by lipid homeostasis-related disturbances^2^. Sterol regulatory element-binding proteins (SREBPs), a family of transcription factors including SREBP-1a, SREBP-1c, and SREBP-2, regulate the expression of genes involved in fatty acid and cholesterol biosynthetic pathways^3^. All three SREBPs are synthesized as inactive precursors located on the endoplasmic reticulum (ER) membrane and are processed to liberate N-terminal halves that function as transcription factors in the nucleus. This proteolytic activation is tightly regulated by the interaction of ER membrane proteins, SREBP cleavage-activating protein (SCAP), and insulin-induced gene (Insig). When intracellular sterol levels are reduced, the SCAP/SREBP complex dissociates from Insig, followed by SCAP binding to the common coated protein II (COPII) proteins Sar1 and Sec23/24, resulting in the incorporation of the SCAP/SREBP complex into COPII-coated vesicles. Consequently, SREBPs enter the Golgi and are processed by site-1 and site-2 proteases^4^. SREBP-1c expression and proteolytic processing are markedly enhanced in the livers of obese mice^5,6^. Furthermore, SREBP-1c overexpression in the liver of transgenic mice causes fatty liver, hypertriglyceridemia, and insulin resistance^7^. Therefore, abnormal SREBP-1c regulation could be central to obesity-related disease development.

Sulforaphane (SFaN) is an isothiocyanate derived from cruciferous vegetables, such as broccoli, cauliflower, and kale. SFaN is produced by myrosinase-mediated hydrolysis of the glucosinolate glucoraphanin. SFaN reportedly acts through nuclear factor erythroid 2-related factor 2 (Nrf2) transcription factor activation to induce phase II detoxification enzymes, such as NAD(P)H-quinone oxidoreductase 1, glutathione *S*-transferases, and heme oxygenase-1^8^. SFaN-mediated Kelch-like ECH-associated protein 1 (Keap1) cysteine residue modification caused the suppression of Nrf2 degradation, and thereby Nrf2 nuclear translocation and target gene expression stimulation^9,10^. SFaN is also reportedly effective in atherosclerosis, diabetes, and obesity treatment^11–13^. However, the underlying mechanisms of these SFaN effects are not fully understood.

Using a luciferase reporter gene assay with the fatty acid synthase (*FAS*) gene promoter region, we have previously reported that several small food ingredient compounds, such as allyl isothiocyanate (AITC), chrysin, and xanthohumol (XN), suppress SREBPs^14–18^. In this study, we identified SFaN as a natural food component that reduced SREBP activities and stimulated SREBP precursor form degradation.

## Results

### Identification of SFaN as a new SREBP inactivator

**Figure 1:**
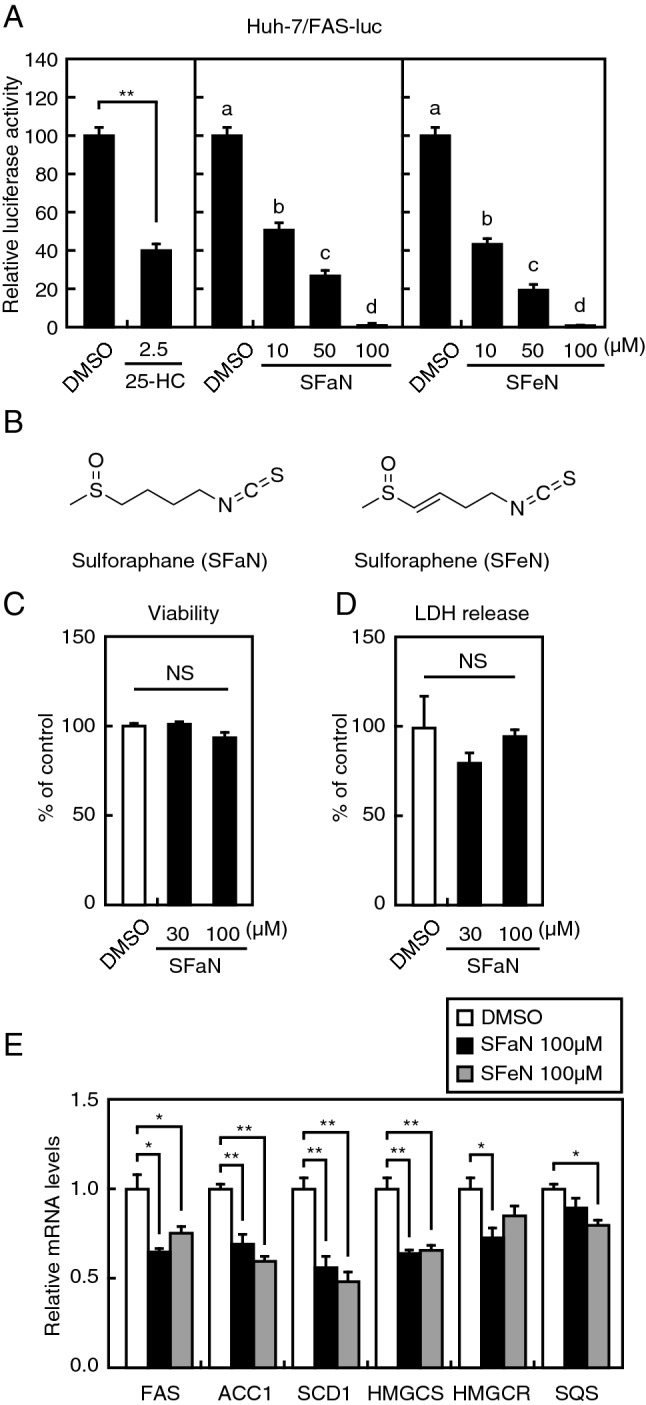
SFaN reduces SREBP activity. (**A**) Huh-7/FAS-luc cells were sterol-depleted by incubation in medium C for 16 h. The cells were then switched to medium C in the presence of the vehicle, 1 μg/ml 25-HC, or the indicated SFaN or SFeN concentrations. After incubation for 24 h, a luciferase assay was performed, and relative luciferase activity was obtained by activity normalization in the presence of the vehicle. All data are represented as the means  Å} S.E. (n = 3). Different superscript letters denote statistical significance (p < 0.05). (**B**) SFaN and SFeN structures. (**C**,**D**) Huh-7 cells were treated with the indicated concentrations of SFaN for 3 h. Afterward, viability (**C**) and LDH (**D**) assays were performed. All data are represented as the means  Å} S.E. (n = 3). NS = not significant. (**E**) Huh-7 cells were sterol-depleted by incubation in medium D for 16 h. The cells were then shifted into medium D in the presence of the vehicle, 100 μM SFaN, or 100 μM SFeN. After incubation for 24 h, the total RNA was isolated from the cells. Real-time quantitative PCR was performed, and relative mRNA levels were obtained by normalization to GAPDH mRNA. All data are represented as the means ± S.E. (n = 3). *, *p* < 0.05; **, *p* < 0.01.

To identify compounds that inhibit SREBP activity, we used a human hepatoma Huh-7 cell line that stably expressed a luciferase reporter gene under the control of an SREBP-driven *FAS* promoter (Huh-7/FAS-luc). To promote SREBP activation, Huh-7/FAS-luc cells were sterol-depleted by incubating them with an HMG-CoA reductase inhibitor (fluvastatin). The cells were then treated with approximately 100 naturally occurring food components, and a luciferase assay was performed to evaluate how they affect SREBP activity. As a positive control, we confirmed that 25-hydroxycholesterol (25-HC), a potent SREBP processing inhibitor, markedly reduced the promoter activity in Huh-7/FAS-luc cells (Fig. 1A). Using this assay system, we showed that SFaN and structural analog sulforaphene (SFeN) significantly lowered luciferase activity (Fig. 1A,B). Next, we investigated the cytotoxic effects of different SFaN concentrations (30 and 100 μM) on Huh-7 and Huh-7/FAS-luc cells by WST-8 and lactate dehydrogenase (LDH) assays. Treatment with SFaN at both concentrations for 3 h did not affect the viability and LDH release from Huh-7 (Fig. 1C,D) and Huh-7/FAS-luc cells (Supplementary Fig. S1), indicating that SFaN cytotoxicity was low. Therefore, 100 μM SFaN was used for further experiments. To determine whether SFaN and SFeN reduce the endogenous SREBP target gene mRNA levels, Huh-7 cells were treated with SFaN or SFeN for 24 h under sterol-depleted conditions induced with lipoprotein-deficient serum. Our real-time quantitative PCR analyses demonstrated that the expression of three SREBP-1 and three SREBP-2 target genes, *FAS*, acetyl-CoA carboxylase 1 (*ACC1*), and stearoyl-CoA desaturase (*SCD1*), and HMG-CoA synthase (*HMGCS*), HMG-CoA reductase (*HMGCR*), and squalene synthase (*SQS*), respectively, decreased with the use of 100 μM SFaN or SFeN (Fig. 1E). These results indicate that SFaN and SFeN suppress SREBP activity and lead to decreased SREBP target gene mRNA levels.

### SFaN reduces SREBP precursor forms prior to the mature SREBP form reduction

**Figure 2:**
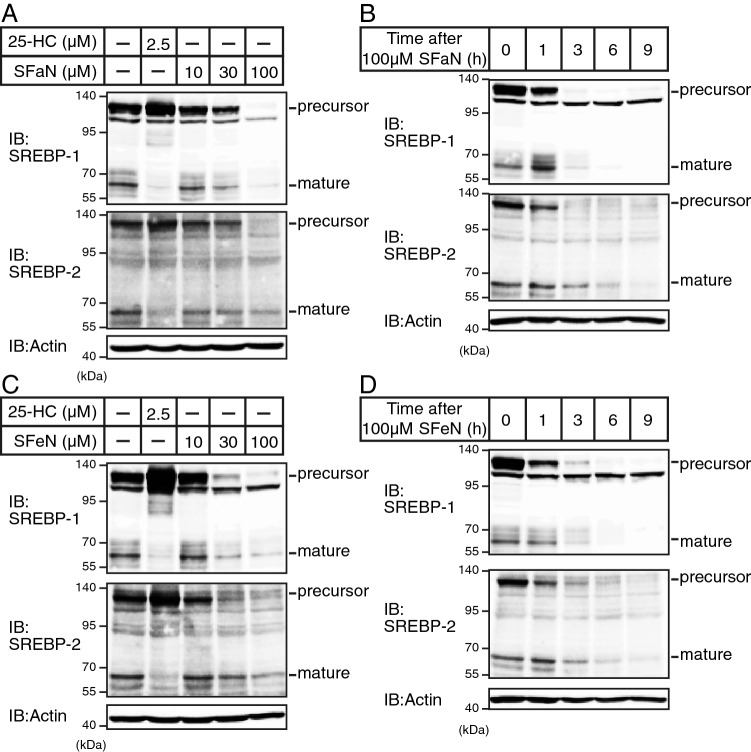
SFaN reduces SREBP precursor and mature form protein levels. (**A**,**C**) Huh-7 cells were sterol-depleted by incubation in medium D for 16 h. The cells were then shifted into medium D in the presence of the vehicle, 1 μg/ml 25-HC, or the indicated concentrations of SFaN (**A**) or SFeN (**C**). After incubation for 3 h, whole-cell extracts underwent immunoblotting (IB) with anti-SREBP-1 (2A4), anti-SREBP-2, or anti-β-actin antibodies. (**B**,**D**) Huh-7 cells were sterol-depleted by incubation in medium D for 16 h. The cells were then shifted into medium D in the presence of 100 μM SFaN (**B**) or SFeN (**D**). After incubation for the indicated periods, whole-cell extracts underwent immunoblotting (IB) with anti-SREBP-1 (2A4), anti-SREBP-2, or anti-β-actin antibodies. Full-length images are presented in Supplementary Fig. S3.

**Figure 3:**
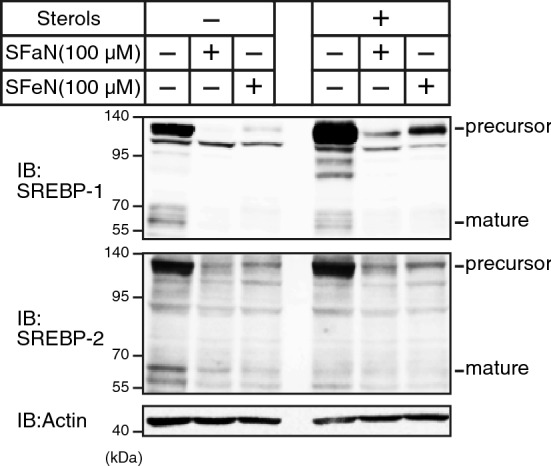
SFaN reduces SREBP precursor forms under cholesterol-enriched conditions. Huh-7 cells were sterol-depleted or enriched by incubation in medium D or medium E for 16 h, respectively, and then treated with 100 μM SFaN or SFeN. After incubation for 3 h, whole-cell extracts underwent immunoblotting (IB) with anti-SREBP-1 (2A4), anti-SREBP-2, or anti-β-actin antibodies. Full-length images are presented in Supplementary Fig. S4.

Next, we investigated whether SFaN and SFeN affect SREBP protein levels. SREBP proteins are synthesized as precursor forms and are then proteolytically cleaved to convert into mature forms under cholesterol-depleted conditions. Hence, Huh-7 cells were depleted of sterols by incubating them in a cholesterol-depleted medium to increase the level of mature SREBP forms. Consistent with previous reports, 25-HC treatment for 3 h reduced the mature SREBP form levels, whereas 10-, 30-, or 100-μM SFaN treatments for 3 h decreased both precursor and mature SREBP forms in a concentration-dependent manner in Huh-7 cells (Fig. 2A). Moreover, SFaN reduced the SREBP precursor, but not the mature SREBP forms after 1 h of treatment, then reduced both precursor and mature SREBP forms after longer treatment durations (Fig. 2B), suggesting that SFaN reduced the SREBP precursor forms prior to the reduction in the mature SREBP forms. Similar results were obtained when cells were treated with SFeN (Fig. 2C,D). These results indicate that the SFaN- and SFeN-related mature SREBP form reduction is attributed to the SREBP precursor form reduction. Next, we investigated whether SFaN and SFeN reduce the SREBP precursor forms under cholesterol-enriched conditions that suppress the proteolytic SREBP processing, thereby increasing the SREBP precursor forms. Figure 3 shows that 3-h SFaN and SFeN treatments reduced the SREBP precursor forms in Huh-7 cells cultured in sterol-supplemented as well as sterol-depleted media. These results indicate that SFaN and SFeN reduce the SREBP precursor forms regardless of the intracellular cholesterol levels.

### SFaN accelerates the SREBP precursor form degradation

**Figure 4:**
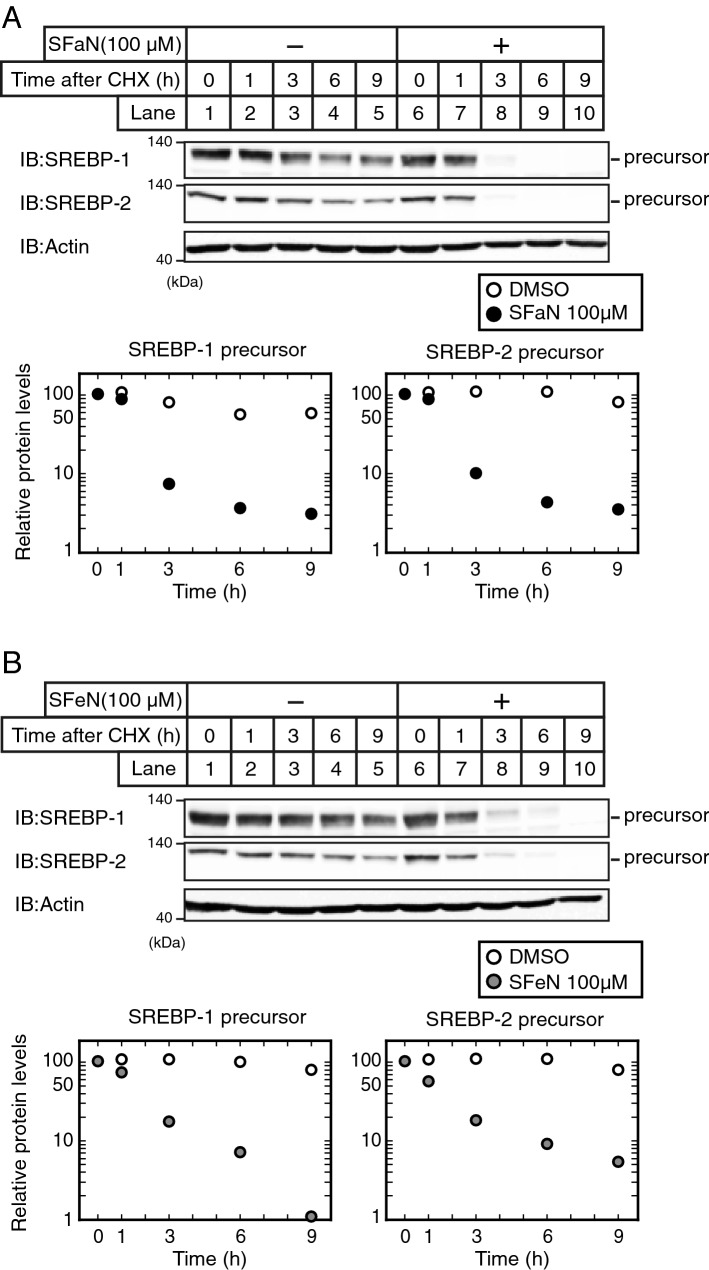
SFaN accelerates SREBP precursor degradation. (**A**,**B**) Huh-7 cells were sterol-supplemented by incubating in medium E for 16 h to increase the SREBP precursor forms. After incubation with 50 μM cycloheximide for 30 min, the cells were shifted into medium E supplemented with 50 μM cycloheximide in the presence of the vehicle, 100 μM SFaN (**A**), or SFeN (**B**). After incubation for the indicated periods, whole-cell extracts underwent immunoblotting (IB) with anti-SREBP-1 (2A4), anti-SREBP-2, or anti-β-actin antibodies. The signals were quantified by Image J and normalized by β-actin signals. Full-length images are presented in Supplementary Fig. S4.

As the SFaN- and SFeN- mediated SREBP precursor form reduction occurred within 1 h (Fig. 2B,D), it was conceivable that their regulatory effects could be observed at the protein and not the mRNA level. To determine whether SFaN and SFeN promote the SREBP precursor form degradation, Huh-7 cells were pretreated with cycloheximide (CHX), a translation inhibitor. In the presence of CHX, the amount of the SREBP precursor forms gradually reduced (Fig. 4A,B, lanes 1–5). SFaN and SFeN accelerated the SREBP precursor form reduction in cells pretreated with CHX (Fig. 4A,B, lanes 6–10), suggesting that SFaN and SFeN promoted the SREBP precursor form degradation.

### SFaN reduces SREBP-1 precursor forms in the SCAP-deficient SRD-13A cell line

**Figure 5:**
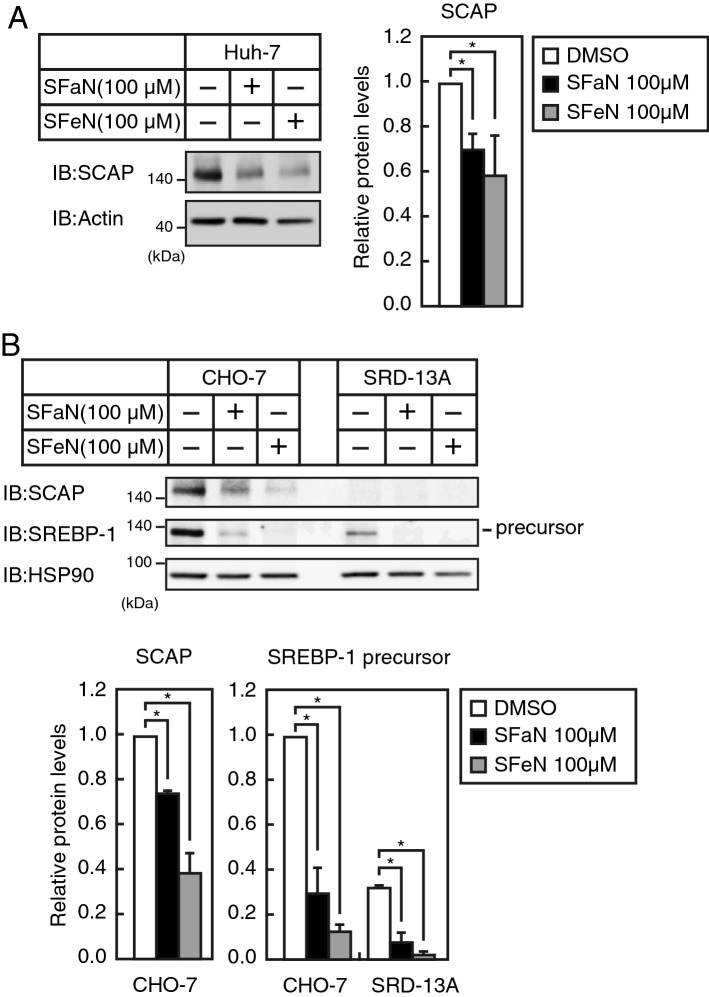
SCAP-independent SREBP precursor form degradation. (**A**) Huh-7 cells were incubated in medium B in the presence of the vehicle, 100 μM SFaN or SFeN for 6 h. Whole-cell extracts underwent immunoblotting (IB) with anti-SCAP or anti-β-actin antibodies. (**B**) CHO-7 and SRD-13A cells were cultured in medium F in the presence of the vehicle, 100 μM SFaN or SFeN for 6 h. Whole-cell extracts underwent immunoblotting (IB) with anti-SCAP, anti-SREBP-1 (2A4), or anti-HSP90 antibodies. The signals (n = 3) were quantified using Image J and normalized to that of β-actin (**A**,**B**). All data are expressed as means ± S.E. *, *p* < 0.05. Full-length images of all replicates are presented in Supplementary Fig. S5.

A previous study has shown that SCAP protein levels directly affect the SREBP precursor stability^19^. In addition, we previously reported that the inhibition of heat shock protein (HSP) 90 destabilizes the SREBP precursor forms preceded by SCAP protein degradation, and this SREBP precursor form destabilization could not be observed in the SCAP-deficient SRD-13A cell line^20^. This line of evidence led us to consider the possible contribution of SCAP in the SFaN- and SFeN-mediated degradation of the SREBP precursor forms. Figure 5A shows that SFaN and SFeN treatments for 6 h reduced the SCAP levels in Huh-7 cells, implying the involvement of the SCAP-dependent pathway in SFaN- and SFeN-mediated SREBP precursor degradation. To investigate whether an SCAP-dependent pathway can contribute to the degradation, we took advantage of an SCAP-deficient cell line SRD-13A derived from Chinese hamster cell line CHO-7^19^. We confirmed that SFaN and SFeN treatments for 6 h reduced the SCAP and SREBP-1 precursor protein levels in CHO-7 cells (Fig. 5B). Consistent with a previous report, SRD-13A cells expressed no detectable SCAP and lower amounts of the SREBP-1 precursor than CHO-7 cells. Importantly, treatment with SFaN and SFeN for 6 h further reduced the levels of the SREBP-1 precursor protein in SRD-13A cells (Fig. 5B), suggesting that in addition to the SCAP-dependent pathway, there is also an SCAP-independent pathway in SFaN- and SFeN-mediated SREBP precursor degradation.

### SFaN accelerates ubiquitin–proteasome-mediated SREBP precursor form degradation

**Figure 6:**
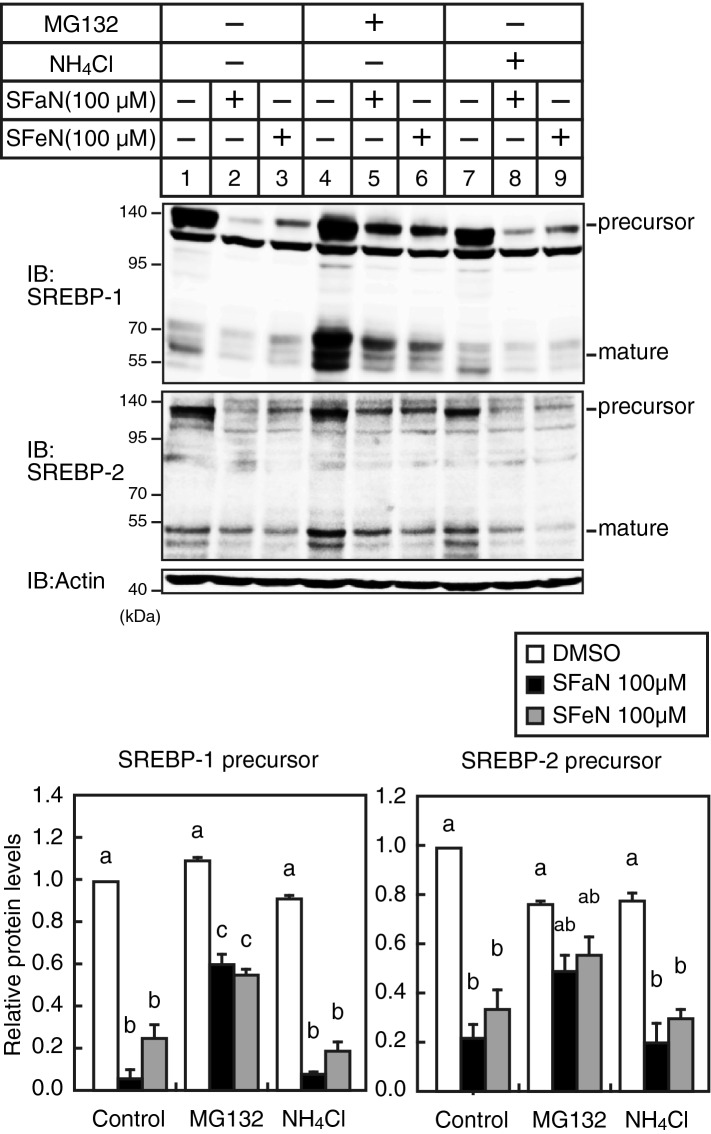
SFaN promotes the ubiquitin–proteasome pathway-mediated SREBP precursor degradation. Huh-7 cells were incubated in medium D for 16 h. After incubation with 10 μM MG132 or 20 mM NH_4_Cl for 30 min, the cells were shifted into medium E supplemented with 10 μM MG132 or 20 mM NH_4_Cl in the presence of the vehicle, 100 μM SFaN or SFeN. After incubation for 3 h, whole-cell extracts underwent immunoblotting (IB) with anti-SREBP-1 (2A4), anti-SREBP-2, or anti-β-actin antibodies. The signal intensities (n = 3) were quantified using Image J and normalized to that of β-actin. The signal intensity of the control group treated with DMSO was arbitrarily defined as one. All data are expressed as means ± S.E. Different superscript letters denote statistical significance (p < 0.05). Full-length images of all replicates are presented in Supplementary Fig. S6.

The ubiquitin–proteasome system and the autophagy-lysosome system are the two major protein degradation pathways in eukaryotic cells. To examine whether SFaN and SFeN regulate the SREBP precursor degradation via these pathways, Huh-7 cells were preincubated with MG132 or NH_4_Cl, a proteasome and lysosome inhibitor, respectively. Consistent with a previous report describing that the mature SREBP forms were degraded through the ubiquitin–proteasome system, the amount of the mature SREBP forms increased in the MG132-, but not in the NH_4_Cl-treated cells (Fig. 6, lanes 1, 4, and 7). We also found that MG132, but not NH_4_Cl treatment increased the SREBP precursor forms. Importantly, the MG132 treatment partly attenuated SFaN- and SFeN-mediated SREBP precursor form reduction (Fig. 6, lanes 1–6), although the NH_4_Cl treatment did not affect them (Fig. 6, lanes 1–3 and 7–9). These results suggest that the degradation of the SREBP precursor form, by SFaN and SFeN, is mediated, in part, through the ubiquitin–proteasome pathway.

### HSP27 is not involved in SFaN accelerated ubiquitin–proteasome pathway-mediated SREBP precursor form degradation

**Figure 7:**
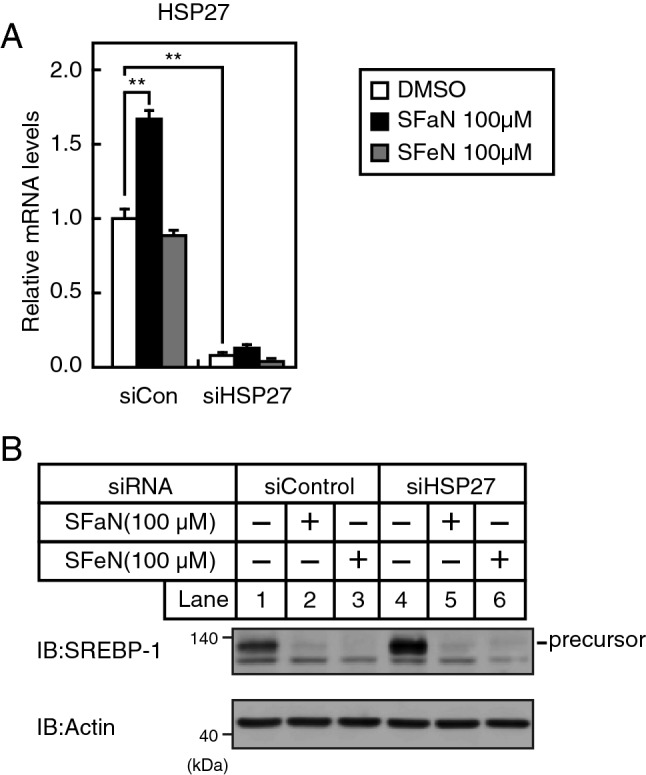
SFaN-mediated SREBP precursor degradation occurs HSP27-independently. (**A**,**B**) Huh-7 cells were transfected with 24 pmol of siRNA (siCon) or HSP27 siRNA and incubated in medium B for 48 h. The cells were then shifted into medium B in the presence of the vehicle, 100 μM SFaN or SFeN for 6 h. Total RNA was isolated from the cells. Real-time quantitative PCR was performed, and relative mRNA levels were obtained by normalization to GAPDH mRNA. All data are represented as the means ± S.E. (n = 3) (**A**). Whole-cell extracts underwent immunoblotting (IB) with anti-SREBP-1 (2A4) or anti-β-actin antibodies (**B**). All data are expressed as mean ± S.E. **, *p* < 0.01. Full-length images of all replicates are presented in Supplementary Fig. S7.

SFaN reportedly enhances proteasome activity through the upregulation of HSP27^21^. To examine whether HSP27 is involved in the SFaN- and SFeN-mediated SREBP precursor form degradation, Huh-7 cells were transfected with siRNA specifically targeting HSP27. Consistent with previous results, SFaN treatment for 6 h induced HSP27 mRNA in siControl-transfected Huh-7 cells (Fig. 7A). It should be noted that SFeN treatment for 6 h did not alter HSP27 mRNA levels, and HSP27expression-knockdown efficiency was confirmed by quantitative RT-PCR (Fig. 7A). Figure 7B shows that the SFaN- and SFeN-mediated SREBP precursor degradation could still be observed in the case of HSP27-knockdown Huh-7 cells as well as siControl-transfected Huh-7 cells, suggesting that HSP27 is not involved in the SFaN- and SFeN-mediated SREBP precursor form degradation effect.

### SFaN accelerates SREBP precursor form ubiquitination

**Figure 8:**
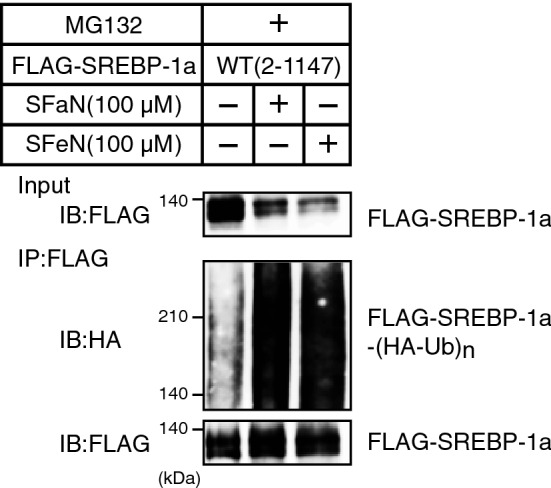
Ubiquitination of SREBP-1a is stimulated by SFaN. Huh-7 cells were transfected with pCMV-SREBP-1a-(2–1147) -3 × FLAG and HA-Ub and cultured in medium B for 24 h. The cells were trypsinized and seeded in 100 mm dishes and cultured in medium B for 24 h. After incubation with 10 μM MG132 for 30 min, the cells were shifted into medium B supplemented with 10 μM MG132 in the presence of vehicle, 100 μM SFaN, or SFeN. After incubation for 3 h, cell lysates were subjected to immunoprecipitation (IP) using an anti-FLAG antibody. The immunoprecipitates were subjected to immunoblotting (IB) with anti-HA or anti-FLAG antibodies. Full-length images are presented in Supplementary Fig. S7.

Next, we investigated whether SFaN and SFeN accelerate the SREBP precursor form ubiquitination. Huh-7 cells were transfected with expression plasmids for full-length human SREBP-1a with a C-terminal FLAG tag [amino acids 2–1147; pCMV-SREBP-1a-(2–1147)-3 × FLAG] and ubiquitin with an N-terminal HA tag (HA-Ub) and cultured in the presence of MG132. Next, SREBP-1a-3 × FLAG was immunoprecipitated with an anti-FLAG antibody and the immunoprecipitates were subjected to immunoblotting using an anti-HA antibody. Although the ladder band signal of ubiquitin-conjugated SREBP-1a was barely detected in the absence of SFaN and SFeN, the SFaN and SFeN treatments intensified it (Fig. 8), suggesting that SREBP-1a was ubiquitinated, stimulated by SFaN and SFeN.

**Figure 9:**
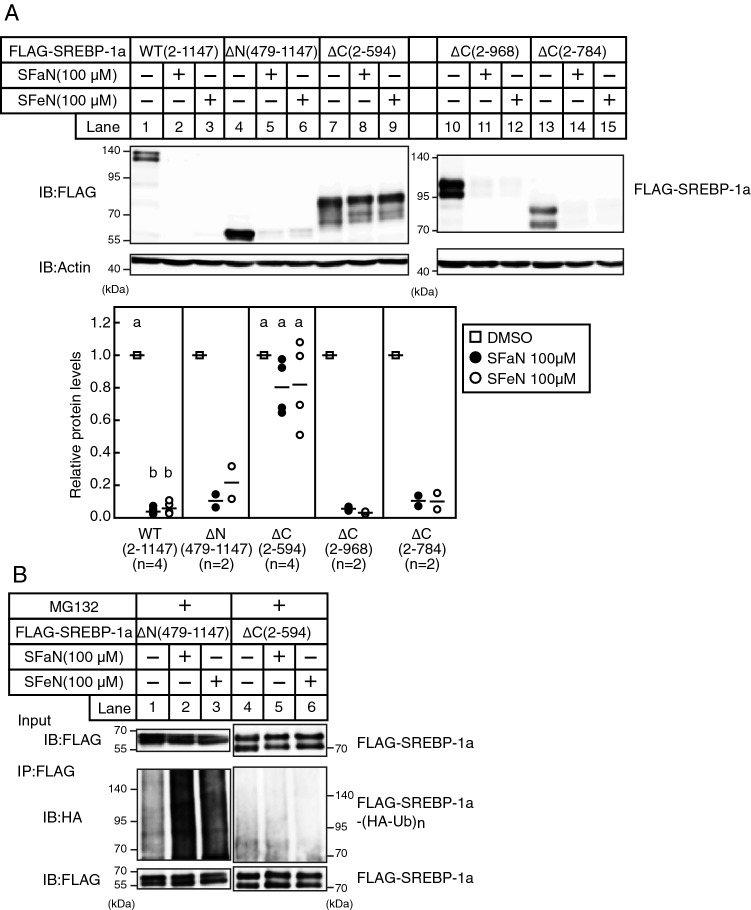
The C-terminal SREBP-1a region is essential for SFaN-mediated SREBP-1a degradation and ubiquitination. (**A**) Huh-7 cells were transfected with pCMV-SREBP-1a-(2–1147)-3 × FLAG, pCMV-SREBP-1a-(479–1147)-3 × FLAG, pCMV-SREBP-1a-(2–594)-3 × FLAG, pCMV-SREBP-1a-(2–968)-3 × FLAG, or pCMV-SREBP-1a-(2–784)-3 × FLAG and cultured in medium B for 24 h. The cells were trypsinized, seeded in six-well plates, and cultured in medium B for 24 h. The cells were then shifted into medium B in the presence of the vehicle, 100 μM SFaN or SFeN. After incubation for 3 h, whole-cell extracts underwent immunoblotting (IB) with anti-FLAG or anti-β-actin antibodies. The signals were quantified by Image J and normalized by β-actin signals, and the signals of the control group treated with DMSO were represented as one (n = 2–4). Different superscript letters denote statistical significance (p < 0.05). Full-length images of all replicates are presented in Supplementary Fig. S8. (**B**) Huh-7 cells were transfected with pCMV-SREBP-1a-(479–1147)-3 × FLAG or pCMV-SREBP-1a-(2–594)-3 × FLAG and HA-Ub and cultured in medium B for 24 h. The cells were trypsinized, seeded in 100 mm dishs, and cultured in medium B for 24 h. After incubation with 10 μM MG132 for 30 min, the cells were shifted into medium B supplemented with 10 μM MG132 in the presence of the vehicle, 100 μM SFaN, or SFeN. After incubation for 3 h, cell lysates were subjected to immunoprecipitation (IP) using an anti-FLAG antibody. The immunoprecipitates were subjected to immunoblotting (IB) with anti-HA or anti-FLAG antibodies. Full-length images are presented in Supplementary Fig. S9.

To identify the SREBP-1a region essential for the SFaN- and SFeN-mediated SREBP-1a precursor form degradation and ubiquitination, we generated two expression plasmids encoding ΔN SREBP-1a [amino acids 479–1147; pCMV-SREBP-1a-(479–1147)-3 × FLAG] and ΔC SREBP-1a [amino acids 2–594; pCMV-SREBP-1a-(2–594)-3 × FLAG]. The ΔN SREBP-1a protein was decreased and the ubiquitinated ΔN SREBP-1a was increased by SFaN and SFeN (Fig. 9A, lanes 4–6 and 9B, lanes 1–3). However, this effect could not be observed in the ΔC versions (Fig. 9A, lanes 7–9 and 9B, lanes 4–6). These results indicate that the C-terminal region is crucial for the SFaN- and SFeN-mediated SREBP-1a precursor form reduction and ubiquitination.

To identify the domain in the SREBP-1a C-terminal region required for the SREBP-1a precursor degradation, Huh-7 cells were transfected with expression plasmids for ΔC SREBP-1a-(2–968) and ΔC SREBP-1a-(2–784), then treated with SFaN or SFeN. ΔC SREBP-1a-(2–968), and ΔC SREBP-1a-(2–784) were degraded by SFaN and SFeN, whereas ΔC SREBP-1a-(2–594) was not (Fig. 9A, lanes 7–15), indicating that the SREBP-1a 595–784 amino acids were essential for the SFaN- and SFeN-mediated SREBP-1a precursor form degradation.

### Nrf2 activation does not contribute to SFaN-mediated SREBP precursor form degradation

**Figure 10:**
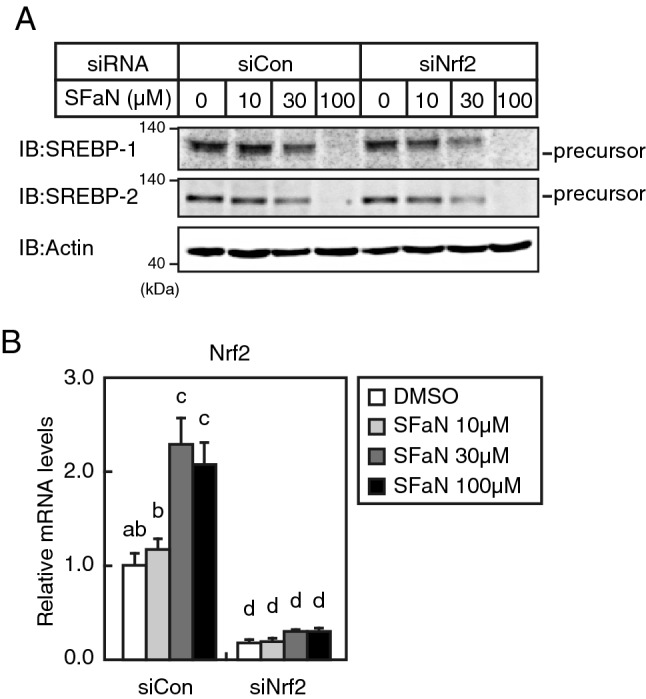
SFaN promotes the Keap1-Nrf2 pathway-independent SREBP precursor degradation. (**A**,**B**) Huh-7 cells were transfected with 24 pmol of siRNA (siCon) or Nrf2 siRNA and incubated in medium B for 48 h. The cells were then shifted into medium B in the presence of the vehicle or the indicated concentrations of SFaN for 3 h. Whole-cell extracts underwent immunoblotting (IB) with anti-SREBP-1 (2A4), anti-SREBP-2, or anti-β-actin antibodies (**A**). The asterisk denotes nonspecific bands. Full-length images are presented in Supplementary Fig. S10. Total RNA was isolated from the cells. Real-time quantitative PCR was performed, and relative mRNA levels were obtained by normalization to GAPDH mRNA. All data are represented as the mean ± S.E. (n = 3) (**B**). Different superscript letters denote statistical significance (p < 0.05).

SFaN reportedly stimulates the expression of genes involved in antioxidant enzymes by inducing Nrf2 nuclear translocation and thereby protects against oxidative stress. To examine whether Nrf2 activation could be involved in the SFaN-mediated SREBP precursor form degradation, Huh-7 cells were transfected with an Nrf2-specific siRNA. The Nrf2 knockdown did not affect the SFaN-mediated SREBP precursor form reduction in Huh-7 cells (Fig. 10A). The Nrf2 expression knockdown efficiency was confirmed by quantitative real-time PCR (Fig. 10B). These results indicate that Nrf2 activation is not required for the SFaN-mediated SREBP precursor form degradation.

### SFaN does not interact directly with SREBP-1a

**Figure 11:**
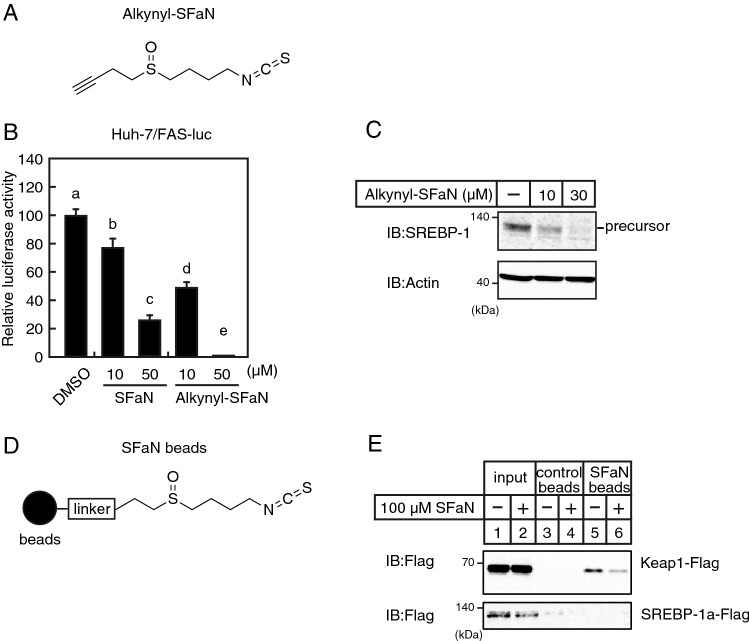
SFaN does not interact with SREBP-1a. (**A**) Structure of alkynyl-SFaN. (**B**) Huh-7/FAS cells were sterol-depleted by incubation in medium C for 16 h. The cells were then switched to medium C in the presence of the vehicle or the indicated concentrations of SFaN or alkynyl-SFaN. After incubation for 24 h, a luciferase assay was performed, and relative luciferase activity was obtained by activity normalization in the presence of the vehicle. All data are represented as the means ± S.E. (n = 3). Different superscript letters denote statistical significance (p < 0.05). (**C**) Huh-7 cells were sterol-depleted by incubation in medium D for 16 h. The cells were then shifted into medium D in the presence of the vehicle or the indicated concentrations of alkynyl-SFaN. After incubation for 3 h, whole-cell extracts underwent immunoblotting (IB) with anti-SREBP-1 (2A4), anti-SREBP-2, or anti-β-actin antibodies. (**D**) Structure of SFaN beads. (**E**) HEK293 cells were transfected with pCMV-SREBP-1a-(2–1147)-3 × FLAG and pCMV-Keap1-3 × FLAG and cultured in medium B for 24 h. Whole-cell extracts underwent pull-down experiments with SFaN beads with or without the addition of 100 μM SFaN and were then subjected to immunoblotting (IB) using an anti-FLAG antibody. Full-length images are presented in Supplementary Fig. S10.

SFaN reportedly interacts directly with Keap1 and thereby activates the transcription factor Nrf2^10,22^. To examine whether SFaN interacts directly with SREBP, SFaN-fixed magnetic beads were generated. An alkyne molecule was introduced into the methylsulfinyl moiety of SFaN (Fig. 11A; alkynyl-SFaN). Alkynyl-SFaN reduced SREBP activity and the levels of SREBP precursor forms to the same extent as SFaN (Fig. 11B,C). Alkynyl-SFaN molecules were fixed to azide-type magnetic beads (FG beads) using click chemistry (Fig. 11D). HEK293 cells were transfected with FLAG-tagged full-length SREBP-1a and Keap1 expression plasmids; then, the cell lysates were exposed to the SFaN beads. The resulting pull-down samples were subjected to immunoblotting using an anti-FLAG antibody. Figure 11E displays that the proteins pulled down with the SFaN beads contained Keap1, whereas the control beads did not (Fig. 11E, upper panel, lanes 3 and 5). Moreover, when the cell lysates were pretreated with additional SFaN, the amount of Keap1 associated with SFaN beads reduced (Fig. 11E, upper panel, lanes 5 and 6), indicating that the SFaN used in the pretreatment competed with the SFaN beads for associating with Keap1. Importantly, the proteins pulled down with SFaN beads did not contain SREBP-1a (Fig. 11E, lower panel), indicating that SFaN did not interact directly with SREBP-1a under these conditions.

## Discussion

Our results demonstrate that naturally occurring isothiocyanates SFaN and SFeN suppressed the *FAS* gene promoter activity and the SREBP target gene expressions. SFaN accelerated the degradation of the SREBP precursor forms. These results suggest that SFaN functions as an SREBP inhibitor, at least in part, by stimulating SREBP precursor form degradation.

SREBP precursor forms reportedly become unstable when the binding partner SCAP is lost^19,23^. We previously reported that HSP90 is required for SCAP protein stability and its inhibition reduced SCAP, promoting SREBP precursor form degradation in mammalian cells^20^. Asano et al. reported that 25-hydroxyvitamin D interacts with SCAP and induces ubiquitin-mediated SCAP degradation, thereby reducing SREBP-2 precursor forms in CHO-K1 cells^24^. In this study, we showed that SFaN and SFeN reduced SCAP protein levels in Huh-7 and CHO-7 cells (Fig. 5). These results suggest that SCAP reduction could be involved in SFaN- and SFeN-mediated degradation of the SREBP precursor forms. We also determined that SFaN and SFeN SCAP independently stimulate the degradation of the SREBP precursor forms (Fig. 5). To the best of our knowledge, ours is the first study describing SCAP-independent SREBP precursor degradation. Further studies are needed to elucidate the contribution of SCAP-dependent and SCAP-independent pathways to the degradation of SREBP precursor by SFaN and SFeN.

We have previously reported that the mature SREBP forms are rapidly degraded by the ubiquitin–proteasome pathway^25^. Sundqvist et al. described that SREBP-1a phosphorylation on Thr426 and Ser430 resulted in Skp1-Cul1-F box protein ubiquitin ligase (SCF^FBW7^) binding, leading to SREBP-1a ubiquitination. However, the SREBP-1a precursor form was not phosphorylated on Thr426 and Ser430 and was insensitive to Fbw7-dependent degradation^26^. In this study, we showed that SFaN stimulates ΔN SREBP-1a (amino acids 479–1147) ubiquitination and degradation, which does not contain these amino acid residues (Fig. 9). Therefore, we considered that SFaN caused SREBP-1a ubiquitination outside the previously reported N-terminal region. Further studies would be required to determine the ubiquitin ligase and amino acid residues involved in the SFaN-mediated SREBP ubiquitination.

Recently, a degradation signal at the SREBP-2 C-terminus has been identified^27^. This signal, comprising seven noncontiguous amino acids, mediates SREBP-2 proteasomal degradation in the absence of SCAP. A degradation signal is also presented at the SREBP-1 C-terminus (amino acids 1034–1071), although the specific amino acids have not been identified. Considering that SFaN-mediated degradation of SREBP-1a also occurs in the absence of SCAP (Fig. 5) and that degradation occurs with ΔC SREBP-1a (amino acids 2–968 and 2–784) (Fig. 9A), we believe that SFaN-mediated SREBP-1a degradation does not require the degradation signal. SFaN-mediated SREBP precursor degradation was partly inhibited by MG132, a proteasome inhibitor (Fig. 6), indicating the involvement of multiple proteolytic systems in the process. Further studies are required to determine whether the degradation signal at the SREBP-2 C-terminus or the proteolytic systems other than the ubiquitin–proteasome system involved in the SFaN-mediated SREBP precursor degradation.

Several small molecules reportedly regulate SREBP activity^4^. Betulin, an abundant compound in birch bark, suppresses proteolytic SREBP processing^28^. Dipyridamole, a phosphodiesterase inhibitor, blocks the ER-to-Golgi transport of the SCAP–SREBP complex independently of its phosphodiesterase inhibitor activity^29^. We have previously reported that XN, the most abundant prenylated flavonoid in hops, impairs the ER-to-Golgi translocation of the SCAP–SREBP complex by binding to Sec23/24 and blocking SCAP/SREBP incorporation into common coated protein II vesicles^18^. In addition, the XN isomer isoxanthohumol (IXN), generated non-enzymatically during the brewing process for beer production, also reduced SREBP activity. However, unlike XN, IXN stimulates the ubiquitin–proteasome-dependent SREBP precursor form degradation^17^. The difference between the SFaN- and IXN-mediated SREBP precursor degradation is their dependence on intracellular cholesterol levels. Although SFaN reduces the SREBP precursor forms regardless of intracellular cholesterol levels (Fig. 3), IXN-mediated reduction is completely abolished under sterol-supplemented conditions^17^. Considering that when the cells are sterol-depleted, the SCAP–SREBP complex is incorporated into COPII-coated vesicles and transported to the Golgi apparatus from the ER, IXN-mediated SREBP precursor degradation might occur during transport or in the Golgi apparatus. However, SFaN-mediated SREBP precursor degradation is presumed to occur on the ER. We have also previously reported that AITC reduces the mature SREBP forms^15^. Although AITC and SFaN are classified isothiocyanates that contain an–N=C=S reactive group, these food factors suppress SREBP activity in different ways, through the reduction of the mature or degradation of the precursor SREBP forms. At present, it remains unclear what causes this difference, although the isothiocyanate group must be required to suppress SREBP activity, and the sulfoxide group, which is not present in AITC, might be involved in SFaN-mediated SREBP precursor degradation. Further studies are required to determine whether other isothiocyanates reduce SREBP activity and could affect precursor or mature SREBPs.

SFaN reported anticancer effects by directly binding to Keap1 and activating the transcription factor Nrf2, thereby detoxifying carcinogenesis^8^. In the present study, we observed the SFaN-mediated degradation of the SREBP precursor forms in Nrf2-knockdown cells (Fig. 10), indicating that this degradation is not due to the SFaN-mediated activation of the Keap1-Nrf2 pathway. This speculation was supported by the fact that degradation was observed within 1 h after the SFaN treatment. Therefore, we considered that SFaN binds to factors other than Keap1 and degrades the SREBP precursor forms. SFaN beads were used to determine whether SFaN binds to SREBP, but no such interaction could be observed (Fig. 11E). Currently, SFaN beads are used to identify novel SFaN-binding proteins directly involved in SFaN-mediated SREBP precursor degradation.

SFaN administration to obesity model animals shows an anti-obesity effect, with a decrease in serum cholesterol and TG levels^30–33^. At present, it remains controversial how SFaN exerts these beneficial effects. One of the promising candidates is the Keap1-Nrf2 pathway^34^. The Keap1-KD-mediated activation of this pathway reportedly reduces weight gain and improves insulin resistance in the short term (8–9 weeks) in HFD-fed mice; however, it conversely causes weight gain and the development of insulin resistance in the long-term in HFD-fed mice^35^. In addition, a decrease in weight gain and improvement in insulin resistance have been observed in Nrf2KO mice^36–38^. These results imply that the anti-obesity effect of SFaN cannot be explained by the Keap1-Nrf2 pathway activation alone. As SREBP-1 activity suppression has been reported to reduce body weight and improve insulin resistance in obese model mice^4,18,28,39^, it is likely that dietary SFaN exerts beneficial effects, at least in part, by the suppression of SREBP-1 activity. Further studies are required to determine whether dietary SFaN suppresses SREBP activity by promoting the SREBP precursor form degradation in the liver of obese mice. SFaN administration also reportedly suppresses adipocyte differentiation, with AMPK activation and decreased peroxisome proliferator-activated receptor γ and CCAAT/enhancer-binding protein α expression in the adipose tissue^31^. More recently, SFaN administration reportedly exerted anti-obesity effects by promoting white adipose tissue browning^40^. These findings suggest that SFaN administration might exert its effects by regulating multiple pathways in multiple organs.

Here, we analyzed the effects of SFaN using human hepatoma Huh-7 cells. We demonstrated that 30 and 100 μM SFaN treatment for 3 h did not affect the viability of Huh-7 cells as well as LDH release from Huh-7 cells, whereas 20–60 μM SFaN treatment for 24 h reportedly decreased the viability of Huh-7 cells^41^, indicating that a longer SFaN treatment period is expected to increase cytotoxicity. Since the degradation of the SREBP precursor forms by SFaN is observed within 3 h of SFaN treatment, it is unlikely that SFaN cytotoxicity is responsible for this effect. We also showed that SFaN treatment with 10 μM SFaN decreased the *FAS* gene promoter activity and 30 μM SFaN promoted the degradation of the SREBP precursor. Further studies are required to verify whether the similar effects are observed in normal hepatocytes and animal models. Considering that the maximum concentration of SFaN metabolites in serum reaches 7.4 μM when 150 mL of test meal containing 100 g florets of super broccoli is consumed by humans^42^, it is unlikely that the consumption of broccoli leads to a SFaN serum levels of 30 μM, and dietary supplements containing highly stable SFaN and SFaN derivatives need to be developed. However, it is believed that compounds derived from food that supplemental intake exist at higher concentrations in the intestinal tract than in the serum. The expression of bitter taste-sensing G protein-coupled receptors [type 2 taste receptors (T2Rs)] is regulated by SREBP-2 in enteroendocrine STC-1 cells^43^. T2Rs are known to sense dietary toxins^44^ and promote cholecystokinin secretion^45^, thereby suppressing food intake. Therefore, it has been hypothesized that T2Rs play a role in limiting toxin absorption. We demonstrated that SFaN promotes SREBP-2 precursor degradation and thereby reduces their activity. Thus, SFaN consumption might downregulate the expression of bitter taste receptors in the intestinal tract and affect the sensing of dietary toxins.

In summary, the present data demonstrate that SFaN reduces SREBP activity by promoting the degradation of SREBP precursors. Furthermore, we showed that C-terminal region is crucial for SFaN- and SFeN-mediated SREBP-1a precursor form degradation. Additional studies are required to elucidate the direct SFaN targets of these effects.

## Methods

### Materials

Cholesterol, 25-HC, CHX, fluvastatin, lipoprotein-deficient serum, MG132, and NH_4_Cl were purchased from Sigma (St Louis, MO, USA). Dulbecco’s modified Eagle’s medium (DMEM) was from Wako (Osaka, Japan). Blasticidin S was from Invitrogen (Carlsbad, CA, USA). SFaN and SFeN were from LKT Laboratories (St Paul, MN, USA).

### Antibodies

Monoclonal anti-SREBP-1 (2A4), anti-SCAP (9D5), and anti-HSP90 (F-8) antibodies were purchased from Santa Cruz (Dallas, TX, USA). Monoclonal anti-FLAG (M2) and anti-β-actin (AC-15) antibodies were purchased from Sigma (St Louis, MO, USA). Monoclonal anti-HA (16B12) antibody was purchased from BioLegend (San Diego, CA, USA). The polyclonal anti-SREBP-2 (RS004) antibody has been previously described^46^. Peroxidase-conjugated affinity-purified donkey anti-mouse IgGs and peroxidase-conjugated affinity-purified donkey anti-rabbit IgGs were purchased from Jackson ImmunoResearch Laboratories (West Grove, PA, USA).

### Media and buffers

Medium A contained DMEM supplemented with 100 U/ml penicillin and 100 μg/ml streptomycin. Medium B was medium A with 10% (v/v) fetal bovine serum (FBS). Medium C was medium A with 10% FBS, 50 μM sodium mevalonate, and 12.5 μM fluvastatin. Furthermore, medium D was medium A with 5% (v/v) lipoprotein-deficient serum, 50 μM sodium mevalonate, and 12.5 μM fluvastatin. Medium E was medium A with 10% FBS, 10 μg/ml cholesterol, and 1 μg/ml 25-HC. Medium F contained DMEM/Ham’s F12 supplemented with 5 μg/ml cholesterol, 1 mM sodium mevalonate, 20 μM sodium oleate, 100 U/ml penicillin, 100 μg/ml streptomycin, and 5% FBS.

### Cell culture

Huh–7 cells and HEK293 cells were obtained from ATCC. CHO-7 cells and SRD-13A cells, an SCAP-deficient line derived from CHO-7 cells, were kindly provided by Debose-Boyd RA (University of Texas Southwestern Medical Center)^19^. Huh-7 cells and HEK293 cells were maintained in medium B. Huh-7/FAS-luc cells (a stable Huh-7 cell line expressing a luciferase reporter driven by an SRE-containing FAS promoter)^15,18,47^ cells were maintained in medium B containing 2 μg/ml blasticidin S. CHO-7 cells and SRD-13A cells were maintained in medium F. SRD-13A cells were originated from the Brown and Goldstein Laboratory^19^. The cells were incubated at 37 °C under a 5% CO_2_ atmosphere.

### Luciferase assays

Huh-7/FAS-luc cells were plated in 12-well plates at a density of 1.0 × 10^5^ cells/well, cultured with medium B for 24 h, and the cells were then switched to medium C for 16 h. After incubation for another 24 h in the absence or presence of 10 μM SFaN or SFeN, 30 μM SFaN or SFeN, or 100 µM SFaN or SFeN, the luciferase activity and protein contents of the cell extracts were measured as described previously^48^. The normalized luciferase values were determined by dividing the luciferase activity by the protein content in the cell extracts quantified using the BCA protein assay (Pierce).

### Cell viability assay and determination of plasma membrane damage

Cell viability and the plasma membrane damage assays were performed as described previously^49^ using Cell Counting Kit-8 (Dojindo) and Cytotoxicity LDH Assay Kit-WST (Dojindo), respectively. Huh-7 or Huh-7/FAS-luc cells were plated in 96-well plates at a density of 2 × 10^4^ cells/well and cultured with medium B for 24 h. After a 3-h incubation in the absence or presence of 30 or 100 μM SFaN, assays were performed following to the manufacturer’s instructions.

### Real-time quantitative PCR

Real-time quantitative PCR was performed as described previously^18^. Total RNA was extracted from Huh-7 cells using ISOGEN (Nippon Gene, Tokyo, Japan) according to the manufacturer’s instructions. RNA was reverse transcribed using a high capacity cDNA reverse transcription kit (Applied Biosystems, Foster City, CA, USA). Real-time quantitative PCR (Taqman probe and SYBR green) analysis was performed on StepOnePlus Real-Time PCR Systems. Expression was normalized to glyceraldehyde-3-phosphate dehydrogenase (GAPDH). The TaqMan ID numbers for genes analyzed are as follows: human (h) FAS, Hs00188012_m1; hSCD1, Hs00748952_s1; hGAPDH, 4352934; The sequences of the primer sets used were as follows: hACC1, 5′-TGGGCCTCAAGAGGATTTGT-3′ and 5′-TCCACTGTTGGCTGATACATAGATG-3′; hHMGCS, 5′-GACTTGTGCATTCAAACATAGCAA-3′ and 5′-GCTGTAGCAGGGAGTCTTGGTACT-3′; hHMGCR, 5′-TACCATGTCAGGGGTACGTC-3′ and 5′-CAAGCCTAGAGACATAATCATC-3′; hHSP27, 5′-CACGAGGAGCGGCAGGACGAG-3′ and 5′-CAGTGGCGGCAGCAGGGGTGG-3′; hNrf2, 5′- TACTCCCAGGTTGCCCACA-3′ and 5′-CATCTACAAACGGGAATGTCTGC-3′; hSQS, 5′-ATGACCATCAGTGTGGAAAAGAAG-3′ and 5′-CCGCCAGTCTGGTTGGTAA-3′.

### Immunoblotting

Immunoblotting was performed as described previously^18^. Cells and mouse liver were lysed in RIPA buffer [50 mM Tris–HCl (pH 8.0), 150 mM NaCl, 1% (v/v) Triton X-100, 0.5% (w/v) deoxycholate, and 0.1% (w/v) SDS] supplemented with a protease inhibitor cocktail. The lysates were subjected to SDS-PAGE, transferred to a polyvinylidene difluoride membrane (Millipore, Billerica, MA, USA), and probed with the antibodies indicated in the figure legends. The immunoreactive proteins were visualized using ECL (GE Healthcare, Milwaukee, WI, USA) or Immobilon (Millipore, Billerica, MA, USA) immunoblotting detection reagents. The signals on the membrane were detected by ImageQuant LAS 4000 mini (GE Healthcare, Milwaukee, WI, USA). Full western blot images are presented in the Supplementary Figures indicated in each figure legend. Blots were cut before incubation with the anti-actin antibody.

### Plasmid constructs

Expression plasmids containing C-terminal FLAG-tagged SREBP-1a-(2–1147), SREBP-1a-(479–1147), SREBP-1a-(2–594), SREBP-1a-(2–784), and SREBP-1a-(2–968) were constructed by ligating NotI-XbaI PCR fragments encoding the corresponding amino acid regions of human SREBP-1a into the pCMV14-3 × FLAG vector (Sigma) in-frame with the C-terminal FLAG tag. The PCR primer sequence sets used were as follows: SREBP-1a-(2–1147), 5′-ATATGCGGCCGCGATGGACGAGCCACCCTTCAGCGAG-3′ and 5′- ATATCTAGAGCTGGAAGTGACAGTGGTCC-3′; SREBP-1a-(479–1147), 5′-ATATGCGGCCGCGATGAGCCGGGGCATGCTGGACCG-3′ and 5′- ATATCTAGAGCTGGAAGTGACAGTGGTCC-3′; SREBP-1a-(2–594), 5′-ATATGCGGCCGCGATGGACGAGCCACCCTTCAGCGAG-3′ and 5′- ATATCTAGAGGCCAGGTCCAGGTCAGCCT-3′; SREBP-1a-(2–784), 5′-ATATGCGGCCGCGATGGACGAGCCACCCTTCAGCGAG-3′ and 5′-ATATCTAGACCATGGGGTACTGAGCACGGA-3′; and SREBP-1a-(2–968), 5′-ATATGCGGCCGCGATGGACGAGCCACCCTTCAGCGAG-3′ and 5′-ATATCTAGAGGCCTTGTCAATGGAGCTGCT-3′. An expression plasmid containing the C-terminal FLAG-tagged full-length Keap1 (1–625) was constructed by inserting PCR fragments encoding the corresponding amino acid regions of human Keap1 into the BamHI site of the pCMV14-3 × FLAG vector in-frame with the C-terminal FLAG tag using In-fusion HD Cloning kit (Takara) following to the manufacturer’s instructions. The PCR primer sequence sets used were as follows: Keap1 (1–625), 5′-CGACTCTAGAGGATCCATGCAGCCAGATCCCAGGCC-3′ and 5′-AGTCAGCCCGGGATCCACAGGTACAGTTCTGCTGGT -3′. The expression plasmid containing HA-ubiquitin (HA-Ub) was a gift from Edward Yeh (Addgene plasmid # 18712)^50^.

### Small interfering RNA (siRNA) experiments

siRNA experiment was performed as described previously^51^. siRNAs for human HSP27 (#6568) and control siRNAs (#6526) were obtained from Cell Signaling Technology. siRNA for human Nrf2 (s9492) was obtained from Ambion; control siRNA (pGL2 luciferase) was obtained from Bonac. Huh-7 cells were transfected with siRNA (24 pmol per six-well plate) using lipofectamine RNAiMAX (Invitrogen) according to the manufacturer’s instructions.

### Immunoprecipitation

Immunoprecipitation was performed as described previously^18^. Huh-7 cells were plated in 100-mm dishes at a density of 2 × 10^6^ cells/dish and cultured with medium B for 24 h. The cells were then transfected with 15 μg of plasmids including 12 μg of pCMV-SREBP-1a-3 × FLAG and 3 μg of HA-Ub. After incubation for 24 h, the cells were trypsinized and seeded in 100 mm dishes and cultured further under the same conditions. After a 24-h incubation, the cells were treated with 10 μM MG132 for 30 min. After incubation for another 3 h in the absence or presence of 100 μM SFaN or SFeN, the cells were harvested and lysed with Nonidet P-40 lysis buffer [50 mM Hepes–KOH (pH 7.6), 100 mM NaCl, 1.5 mM MgCl_2_, 1% (v/v) Nonidet P-40, 1 mM DTT] supplemented with a protease inhibitor cocktail. The cell lysate was passed through a 25-gauge needle 20 times, rotated at 4 °C for 1.5 h, and centrifuged at 20,000×*g* for 30 min. The supernatant was rotated overnight with anti-FLAG M2 Affinity gel (Sigma) at 4 °C, and the pelleted gel was washed 3 times with Tris-buffered saline (TBS). The bound proteins were eluted with 3 × FLAG peptide and were subjected to immunoblotting.

### Synthesis of alkynyl-SFaN (4-((4-isothiocyanatobutyl)sulfinyl)but-1-yne, 1) (Supplementary Fig. S2)

*N*-(4-mercaptobutyl)phthalimide (**2**) was prepared according to the reported procedure by Ren’s group^52^.

Under Ar atmosphere, to a solution of thiol **2** (900 mg, 3.8 mmol) in THF (13 mL) were added NaH (60%, 250 mg, 6.2 mmol) and 4-bromobut-1-yne (420 µL, 4.5 mmol) at room temperature. After stirring for 2 h at room temperature, the reaction mixture was poured into water at 0 °C and the resulting mixture was extracted with ethyl acetate. The combined organic layer was washed with brine, dried over anhydrous magnesium sulfate, and concentrated in vacuo. The residue was subjected to silica gel column chromatography (Hex/EtOAc = 10:1) to give *N*-(4-(but-3-ynylthio)butyl)phthalimide (**3**, 400 mg, 36%).

Compound **3**: ^1^H NMR (400 MHz, CDCl_3_): δ (ppm) = 1.64 (2H, m), 1.79 (2H, m), 2.02 (1H, t, *J* = 2.8 Hz), 2.47 (2H, dt, *J* = 2.8, 7.2 Hz), 2.61 (2H, t, *J* = 7.2 Hz), 2.68 (2H, t, *J* = 7.2 Hz), 3.70 (2H, t, *J* = 7.2 Hz), 7.71 (2H, dd, *J* = 3.2, 5.6 Hz), 7.84 (2H, dd, *J* = 3.2, 5.6 Hz).

Under Ar atmosphere, to a solution of phthalimide **3** (300 mg, 1.0 mmol) in MeOH (5 mL) was added hydrazine monohydrate (78 mg, 1.5 mmol) and the mixture was refluxed for 3 h. Then conc. H_2_SO_4_ (200 µL) was added to the reaction mixture, which was refluxed for further 1 h. After cooling down to room temperature, water (6.5 mL) was added to the mixture, and the resulting precipitate was removed by filtration. Solvent was evaporated in vacuo to give crude 4-(but-3-yn-1-ylthio)butan-1-amine (80 mg), which was used in the next reaction without further purification. This crude amine (80 mg) was dissolved in CH_2_Cl_2_ (1.7 mL) and treated with di-2-pyridyl thionocarbonate (120 µg, 0.52 mmol), followed by 5% aqueous NaOH solution (1.2 mL). The mixture was stirred for 4 h at room temperature, then diluted with H_2_O, and extracted with CHCl_3_. The combined organic layer was dried over anhydrous magnesium sulfate, and concentrated in vacuo. The residue was subjected to silica gel column chromatography (Hex/EtOAc = 10:1–3:1) to give but-3-yn-1-yl(4-isothiocyanatobutyl)sulfane (**4**, 60 mg, 29% in two steps) as a pale yellow oil.

Compound **4**: ^1^H NMR (400 MHz, CDCl_3_): δ (ppm) = 1.70–1.86 (4H, m), 2.05 (1H, t, *J* = 2.4 Hz), 2.49 (2H, dt, *J* = 2.4, 7.2 Hz), 2.62 (2H, t, *J* = 7.2 Hz), 2.70 (2H, t, *J* = 7.2 Hz), 3.56 (2H, t, *J* = 6.4 Hz).

Under Ar atmosphere, to a solution of sulfide **4** (60 mg, 0.30 mmol) in CH_2_Cl_2_ (2.7 mL) was slowly added m-chloroperoxybenzoic acid (77%, 74 mg, 0.33 mmol) at – 78 °C. The solution was After stirring for 1 h at − 78 °C, saturated sodium bicarbonate solution and 10% aqueous sodium thiosulfate solution were added to the reaction mixture and the mixture was extracted with CH_2_Cl_2_. The combined organic layer was washed with saturated sodium bicarbonate solution and brine successively, dried over anhydrous magnesium sulfate, and concentrated in vacuo. The residue was subjected to silica gel column chromatography (Hex/EtOAc = 3:1–1:2) to give 4-((4-isothiocyanatobutyl)sulfinyl)but-1-yne (**1**, 25 mg, 39%) as a colorless oil.

Compound **1**: IR (film): ν_max_ (cm^-1^) = 2179, 2100, 1024. ^1^H NMR (400 MHz, CDCl_3_): δ (ppm) = 1.84–2.01 (4H, m), 2.09 (1H, t, *J* = 2.4 Hz), 2.70–2.86 (4H, m), 2.89 (2H, t, *J* = 6.8 Hz), 3.61 (2H, t, *J* = 6.4 Hz). HRMS (ESI-TOF): m/z calcd. for C_9_H_13_NNaOS_2_ [M + Na]^+^ 238.0331, found 238.0340.

### Alkynyl-SFaN-immobilized beads (SFaN beads) preparation

SFaN beads preparation was performed according to a previously published procedure^53^, with minor modifications. Immobilization was performed with FG beads (Tamagawa Seiki, Nagano, Japan) according to the manufacturer’s instructions, with modifications. Azide beads (1 mg) were incubated with 62.5 μM Tris[(1-benzyl-1H-1,2,3-triazol-4-yl)methyl]amine, 1.25 mM CuSO_4_, 1.25 mM ( +)-Sodium l-ascorbate, and 125 μM SFaN-Alkine-derivative in tert-butyl alcohol/DMSO (4:1) for 16 h at room temperature. The beads were collected by centrifugation, washed three times with tert-butyl alcohol/DMSO/H_2_O (4:1:5), then washed three times with MeOH/H_2_O (1:1) and stored in MeOH/H_2_O (1:1) at 4 °C.

### Determination of SFaN binding proteins

HEK293 cells were plated in 6 well plate at a density of 5 × 10^5^ cells/dish and cultured with medium B for 24 h. The cells were then transfected with 1 μg of pCMV-SREBP-1a-(2–1147) -3 × FLAG or pCMV-Keap1-3 × FLAG plasmid. After incubation for 24 h, cells were lysed in NP-40 buffer [25 mM Tris–HCl (pH 8.0), 100 mM NaCl, 0.5% (v/v) NP-40, 10 mM MgCl_2_, 2 mM EDTA and 10% (v/v) glycerol] supplemented with a protease inhibitor cocktail. The lysed cells were centrifuged at 20,000×*g*. The supernatant was collected and incubated with the SFaN beads under conditions with or without 100 μM SFaN for 1.5 h at 4 °C. After collecting the beads magnetically and washing them four times with wash buffer [25 mM Tris–HCl (pH 8.0), 150 mM NaCl, 0.5% (v/v) NP-40, 10 mM MgCl_2_, 2 mM EDTA, and 10% (v/v) glycerol], SDS sample buffer was added to the beads, and then the suspensions were heated at 96 °C for 5 min. The elution samples were subjected to immunoblotting.

### Statistical analysis

All data are represented as mean ± S.E. Statistical analysis was performed using the Ekuseru-Toukei Ver.2.0 (Social Survey Research Information). Comparisons between treatments were made by a Student’s *t test* for two groups. One-way ANOVA followed by the Bonferroni procedure was used to compare more than two groups. Differences were considered significant at *P* < 0.05.

## Supplementary Information

Supplementary Figures.

## Data Availability

The datasets used and/or analyzed during the current study are available from the corresponding author on reasonable request.
