# Supplementary material for: Sulforaphane suppresses the activity of sterol regulatory element-binding proteins (SREBPs) by promoting SREBP precursor degradation
Source: Sci Rep. 2022 May 24;12:8715. doi: 10.1038/s41598-022-12347-6 (PMC9130306; doi:10.1038/s41598-022-12347-6)
Supplement: Supplementary file 1 — Supplementary Figures. [file 41598_2022_12347_MOESM1_ESM.pdf]

Sulforaphane Suppresses the Activity of Sterol Regulatory Element-binding Proteins (SREBPs) by Promoting SREBP Precursor Degradation

Shingo Miyata[1], Manami Kodaka[2], Akito Kikuchi[2], Yuki Matsunaga[2], Kenta Shoji[2], Yen-Chou Kuan[1, #a], Masamori Iwase[1], Keita Takeda[3], Ryo Katsuta[3], Ken Ishigami[3], Yu Matsumoto[2], Tsukasa Suzuki[2], Yuji Yamamoto[2], Ryuichiro Sato[1, *], and Jun Inoue[2, *]

From the [1]Department of Applied Biological Chemistry, Graduate School of Agricultural and Life Sciences, The University of Tokyo, Tokyo 113-8657, Japan
[2]Department of Agricultural Chemistry, Faculty of Applied Biosciences, Tokyo University of Agriculture, Tokyo 156-8502, Japan
[3]Department of Chemistry for Life Sciences and Agriculture, Tokyo University of Agriculture, Tokyo 156-8502, Japan
[#a]Current Address: Department of Horticulture and Landscape Architecture, College of Bioresources and Agriculture, National Taiwan University, Taipei, Taiwan

[*] Correspondence and requests for materials should be addressed to R.S (email: roysato@g.ecc.u-tokyo.ac.jp) and J.I. (email: ajinoue@mail.ecc.u-tokyo.ac.jp)

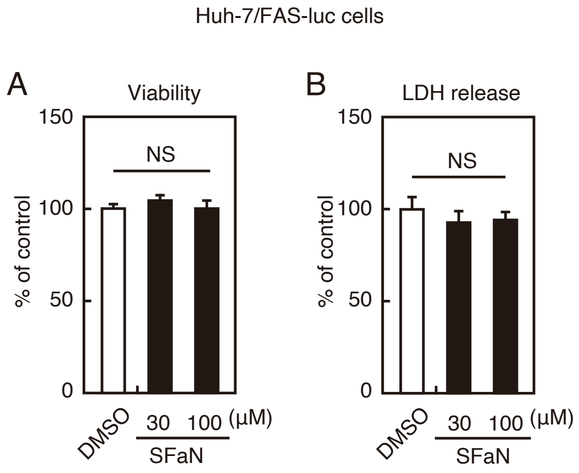

Supplementary Figure S1.

Effect of SFaN on Huh-7/FAS-luc cell viability and LDH release

A and B. Huh-7/FAS-luc cells were treated with the indicated concentrations of SFaN for 3 h, followed by viability (A) and LDH (B) assays. All data are represented as the means ± S.E. (n = 3). NS = not significant.

Fig. S2. Synthesis of alkynyl-SFaN (**1**)

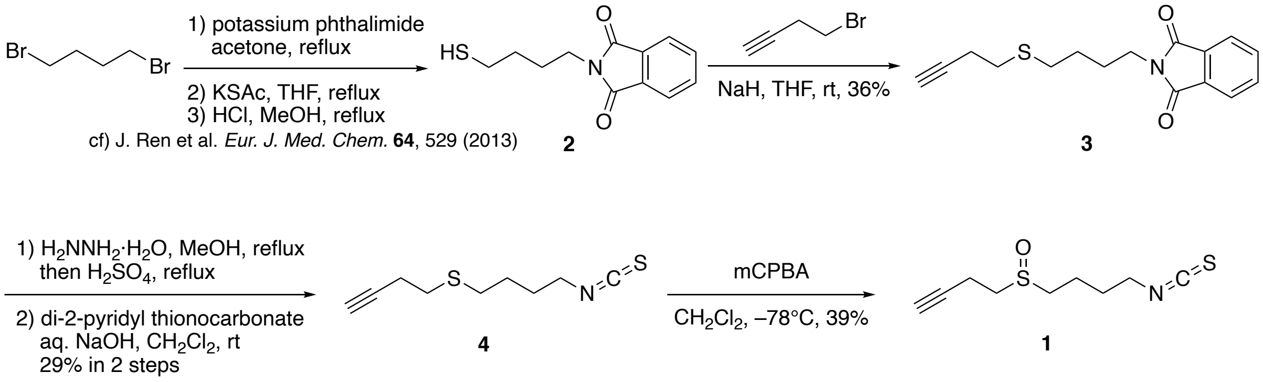

Fig. 2A

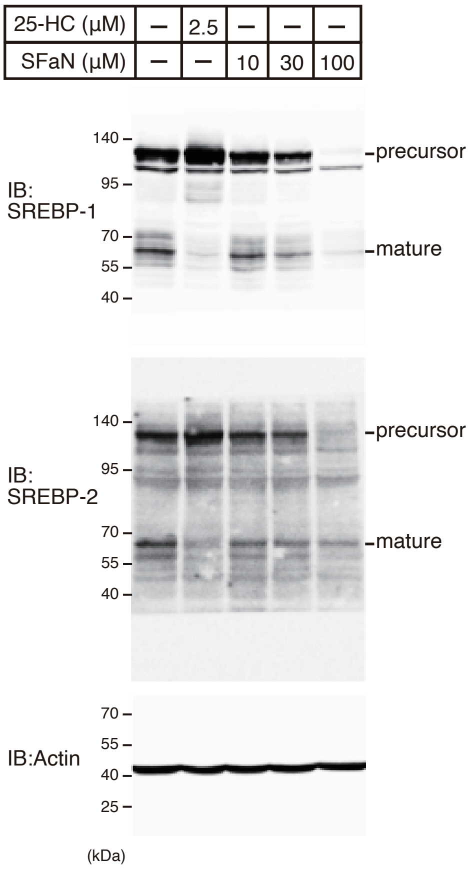

Fig. 2B

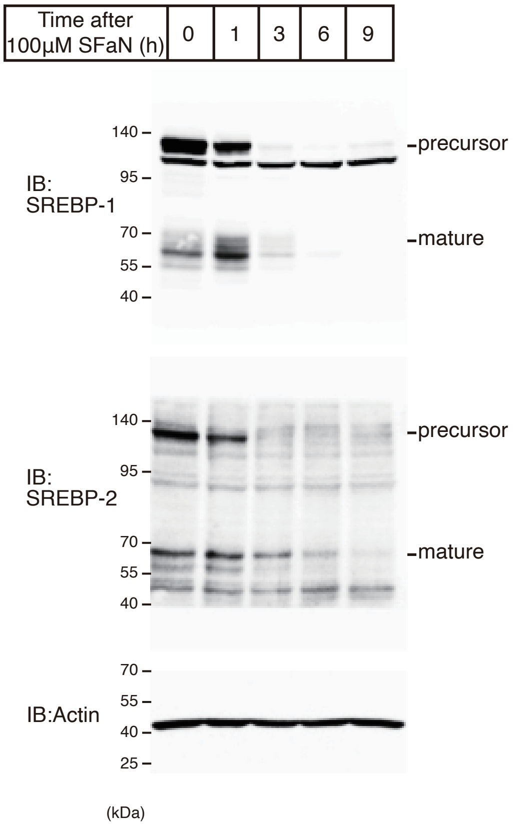

Fig. 2C

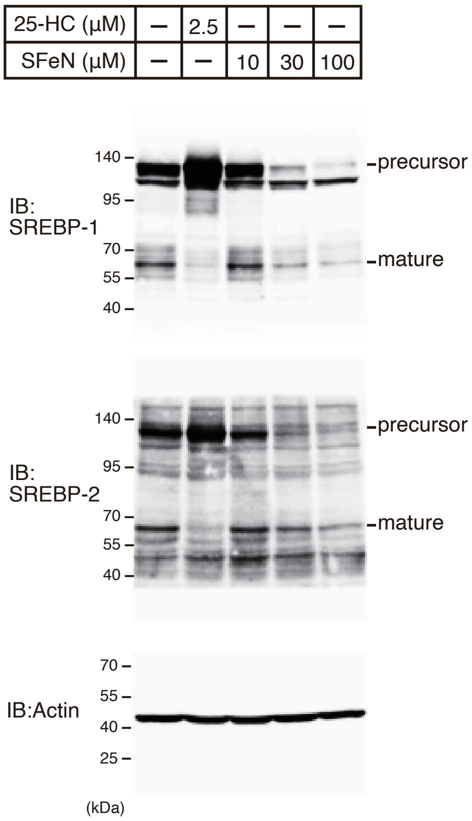

Fig. 2D

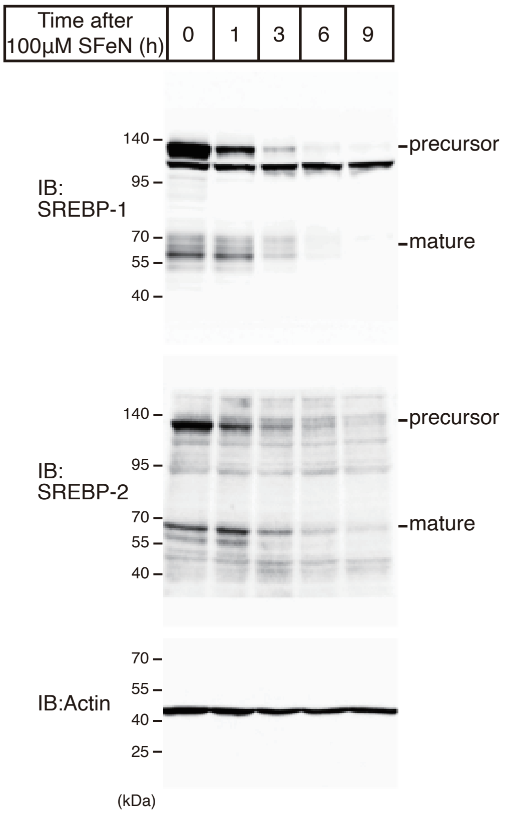

Supplementary Figure S3. ***Full western blot images of Figure 2***

Fig. 3

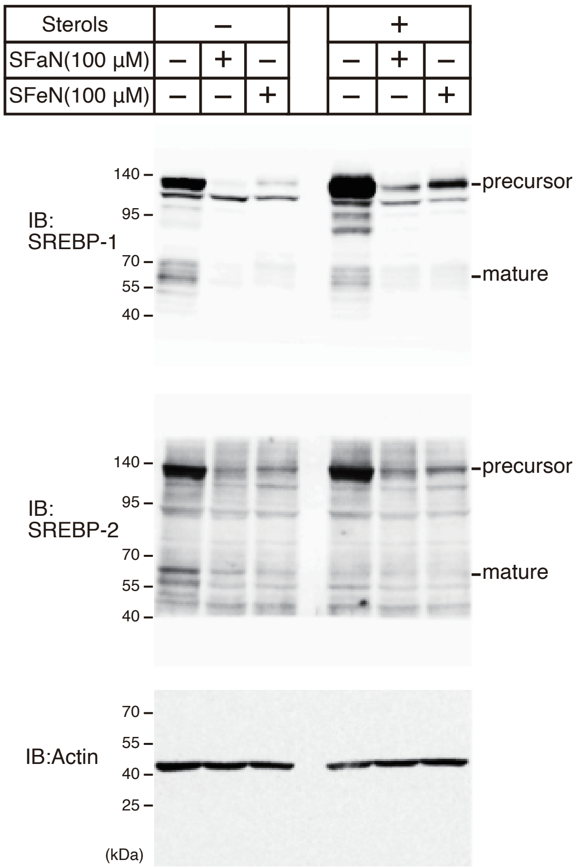

Fig. 4A

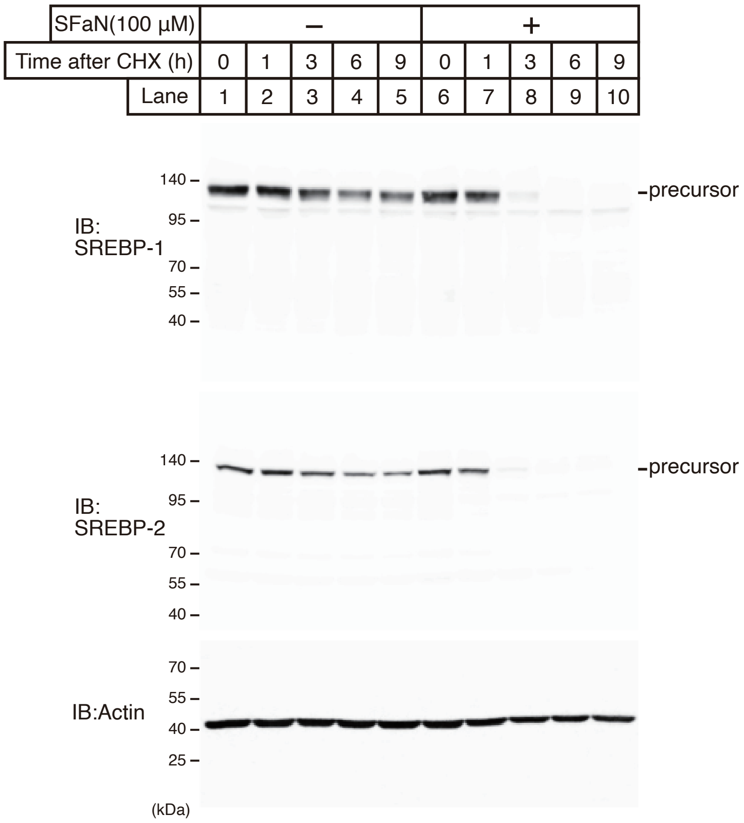

Fig. 4B

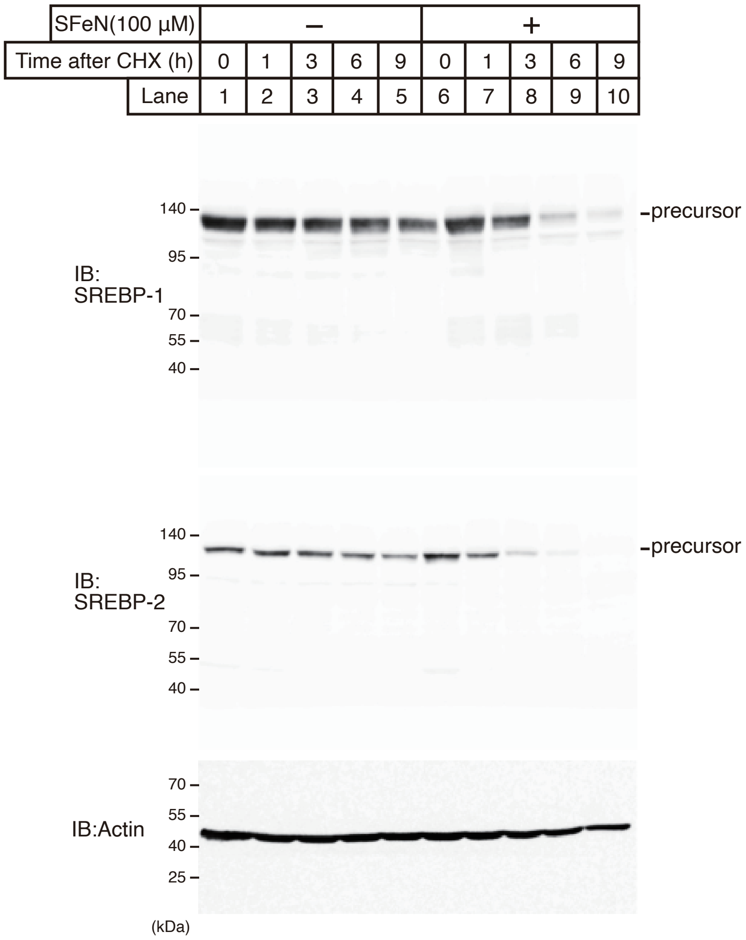

Supplementary Figure S4. ***Full western blot images of Figure 3 and 4***

Fig. 5A, Experiment #1

	Huh-7		
SFaN(100 μM)	–	+	–
SFeN(100 μM)	–	–	+

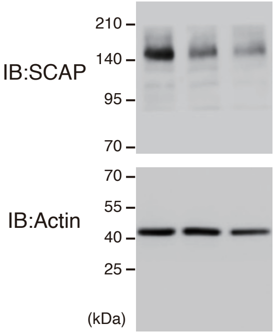

Fig. 5B, Experiment #1

	CHO-7				SRD-13A		
SFaN(100 μM)	–	+	–		–	+	–
SFeN(100 μM)	–	–	+		–	–	+

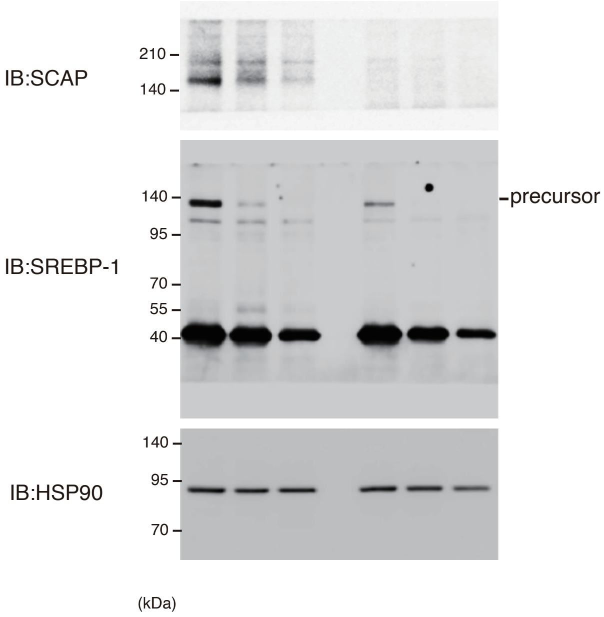

Fig. 5A, Experiment #2 and #3

	Huh-7		
SFaN(100 μM)	–	+	–
SFeN(100 μM)	–	–	+

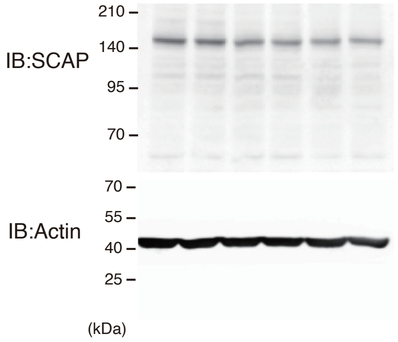

Fig. 5B, Experiment #2 and #3

	CHO-7				CHO-7		
SFaN(100 μM)	–	+	–		–	+	–
SFeN(100 μM)	–	–	+		–	–	+

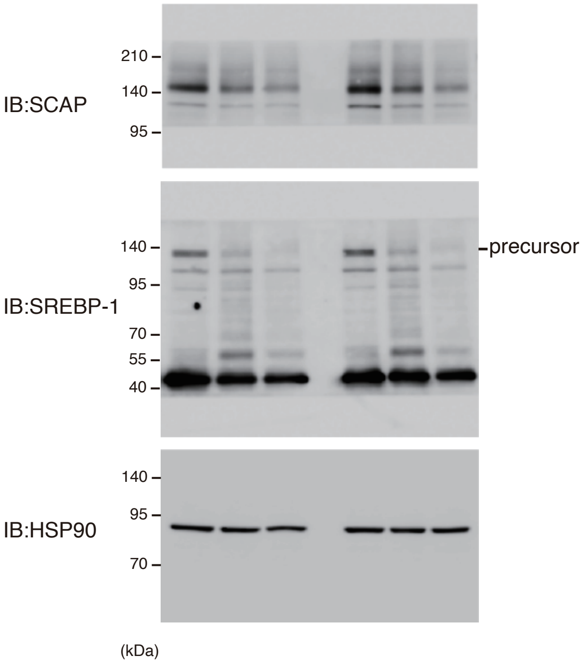

Fig. 5B, Experiment #2 and #3

	SRD-13A				SRD-13A		
SFaN(100 μM)	–	+	–		–	+	–
SFeN(100 μM)	–	–	+		–	–	+

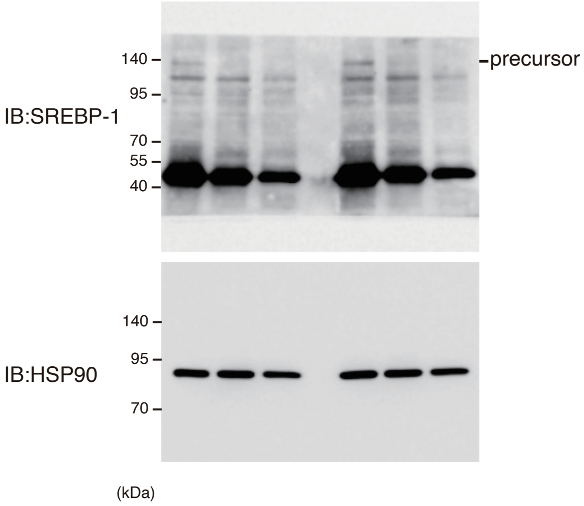

Supplementary Figure S5. ***Full western blot images of Figure 5***

Fig. 6, Experiment #1

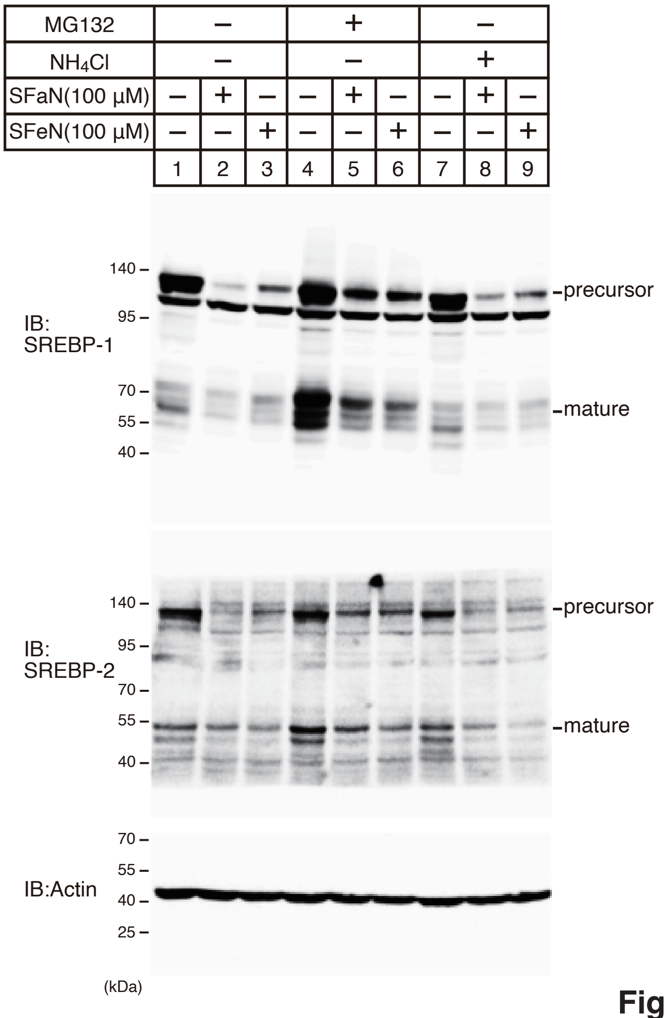

Fig. 6, Experiment #2

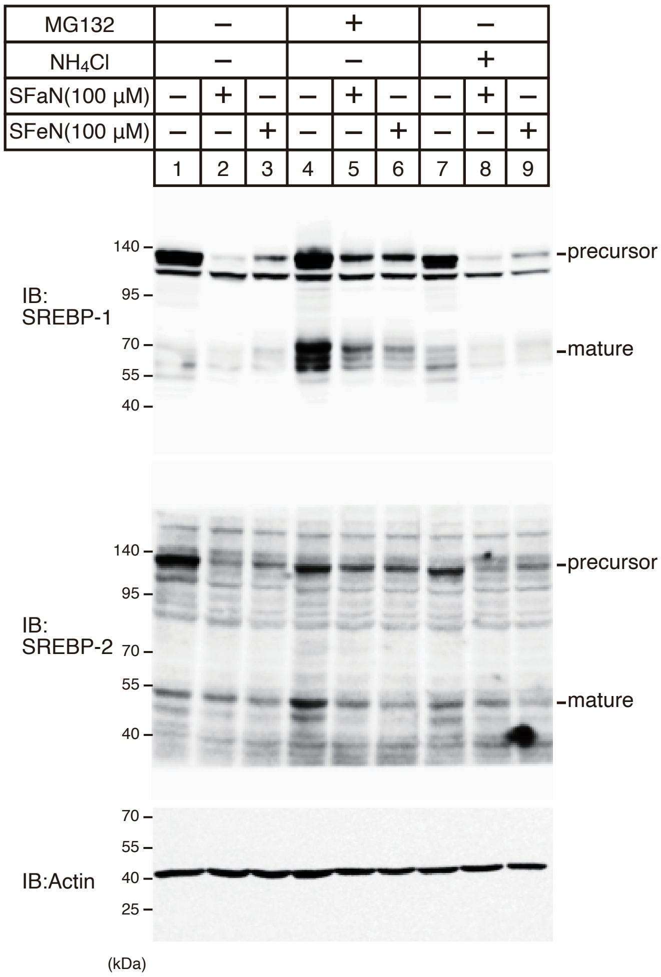

Fig. 6, Experiment #3

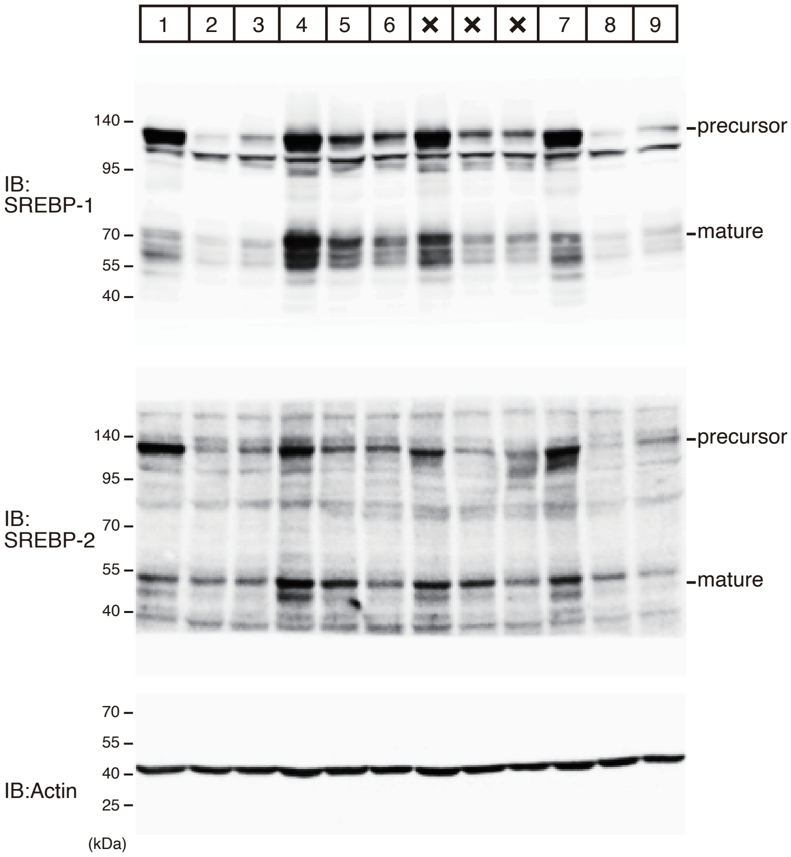

Supplementary Figure S6. ***Full western blot images of Figure 6***

Fig. 7B

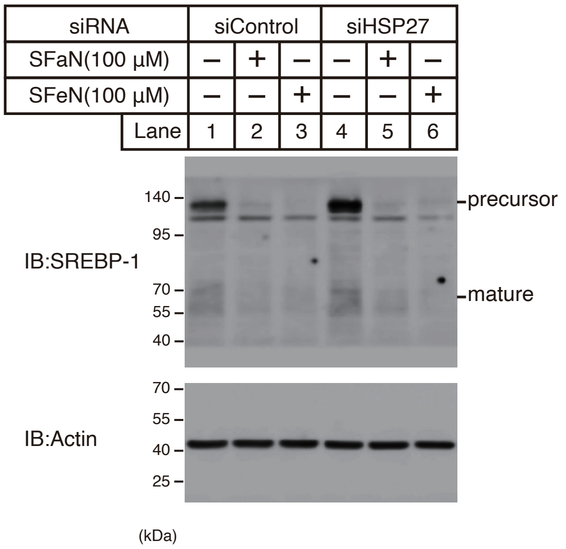

Fig. 8

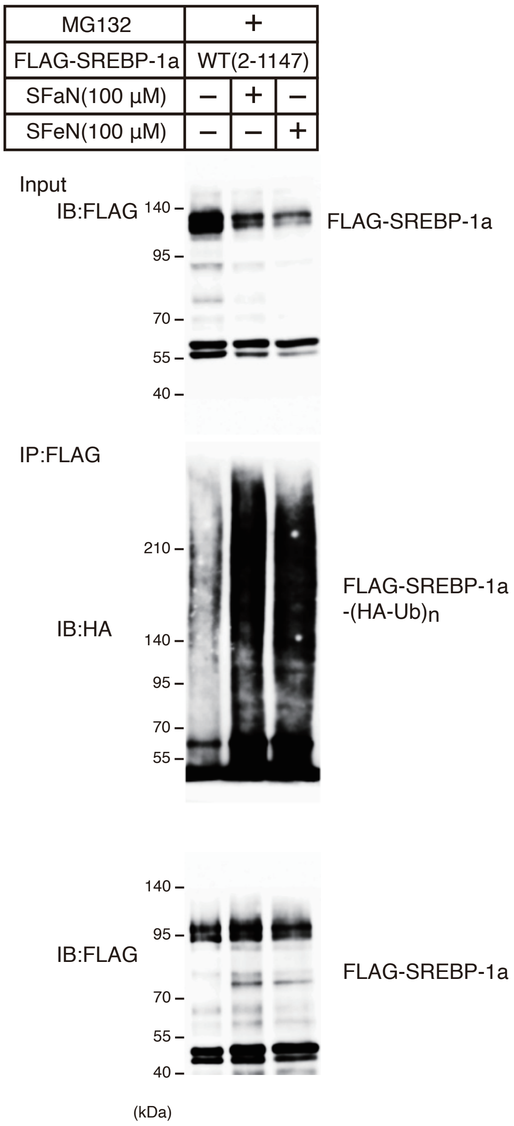

Supplementary Figure S7. ***Full western blot images of Figure 7 and 8***

Fig. 9A

FLAG-SREBP-1a	WT(2-1147)			ΔN(479-1147)			ΔC(2-594)				ΔC(2-968)			ΔC(2-784)		
SFaN(100 μM)	–	+	–	–	+	–	–	+	–		–	+	–	–	+	–
SFeN(100 μM)	–	–	+	–	–	+	–	–	+		–	–	+	–	–	+
Lane	1	2	3	4	5	6	7	8	9		10	11	12	13	14	15

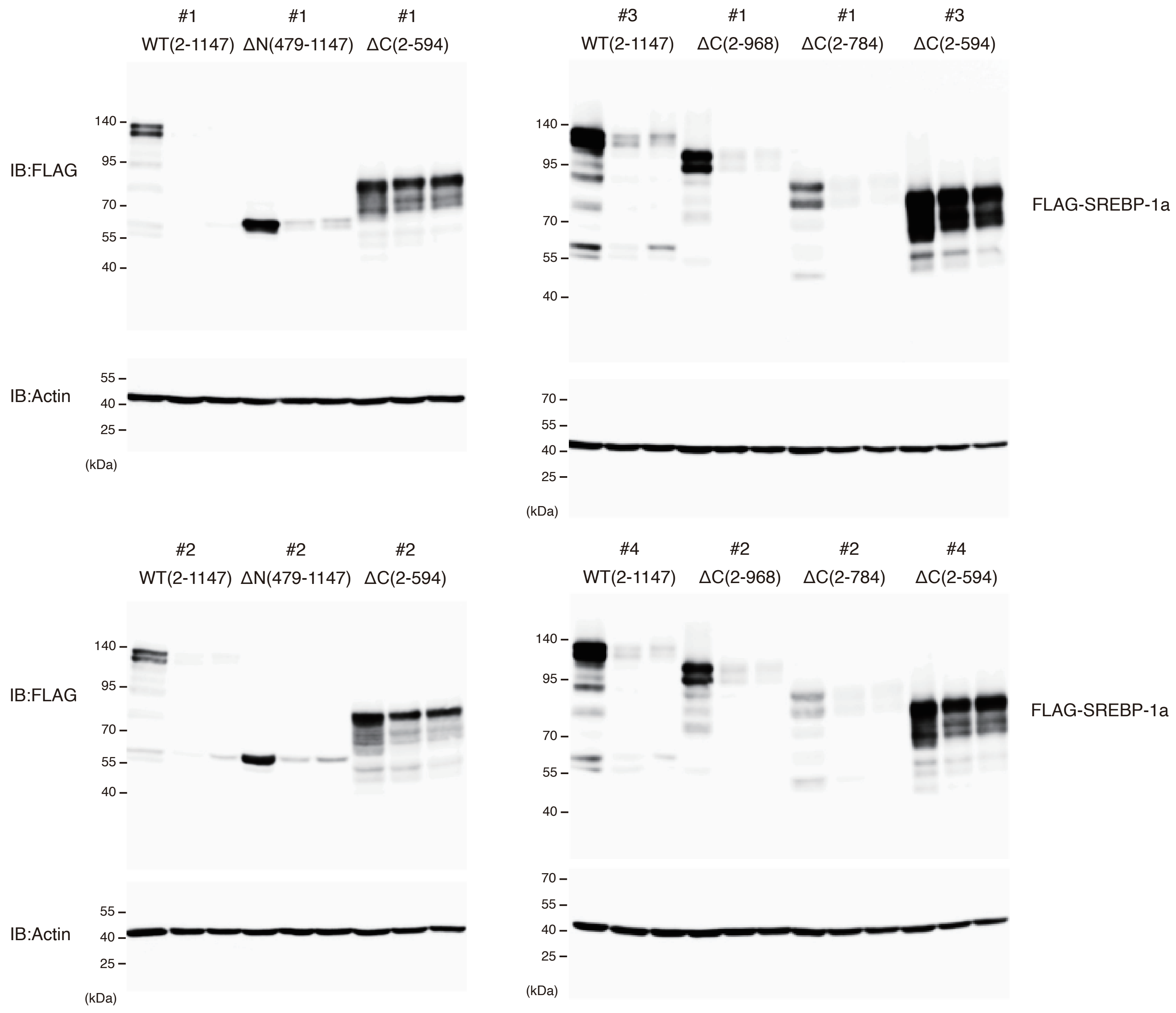

Supplementary Figure S8. ***Full western blot images of Figue 9A***

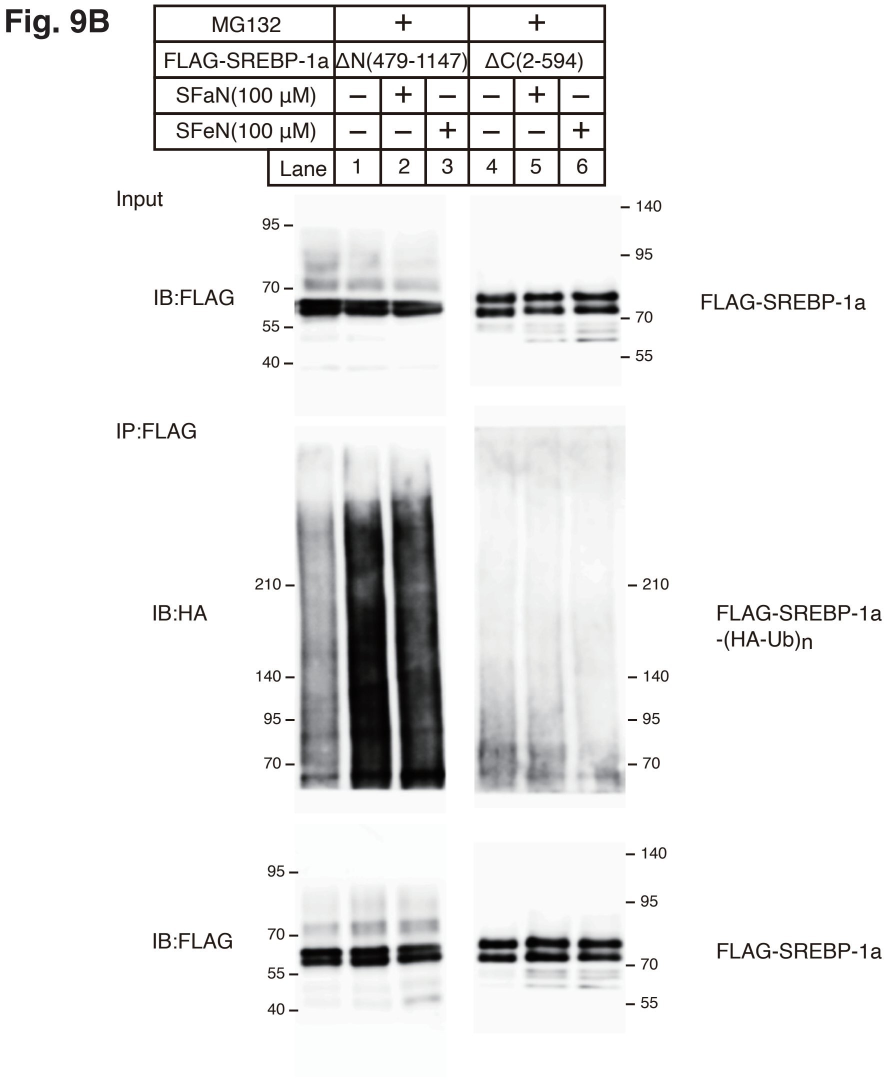

Supplementary Figure S9. ***Full western blot images of Figue 9B***

Fig. 10A

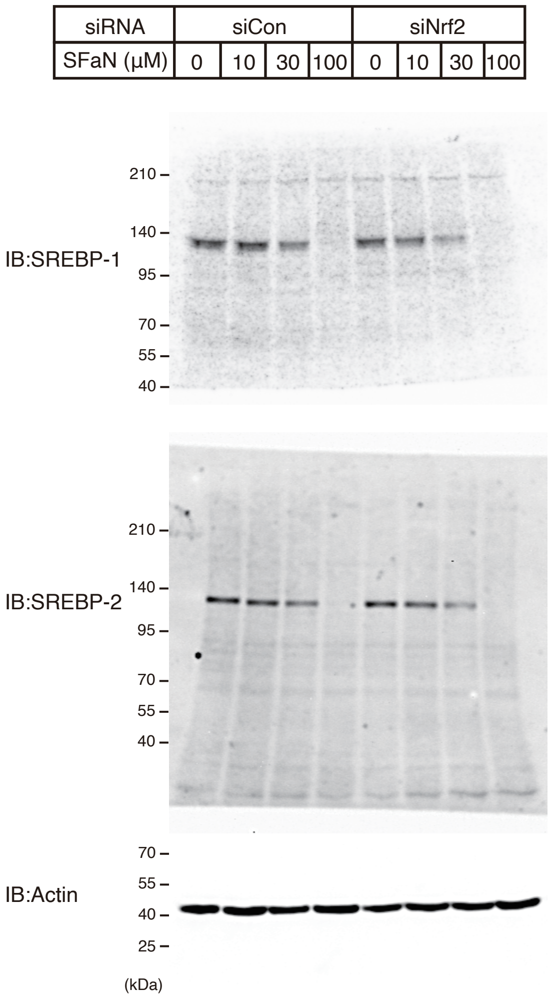

Fig. 11C

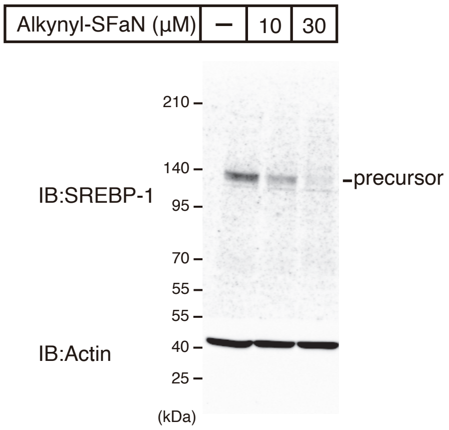

Fig. 11E

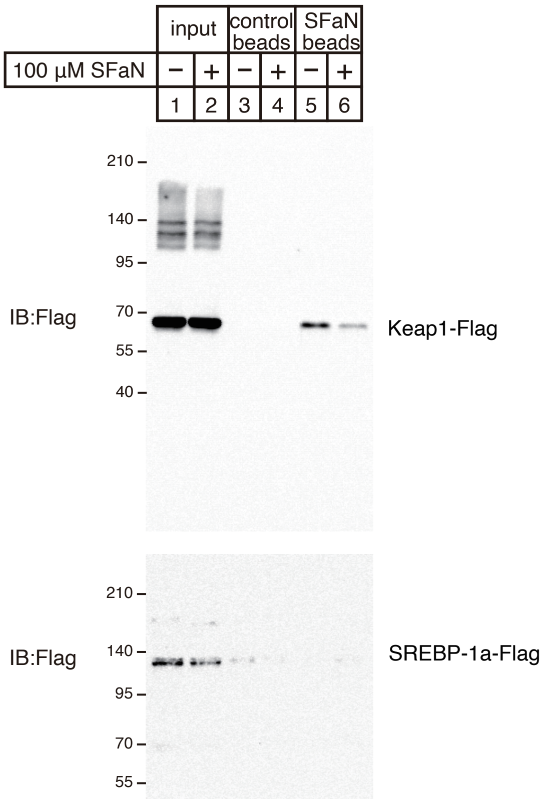

Supplementary Figure S10. ***Full western blot images of Figure 10 and 11***
